# Fe_3_O_4_@Sap/Cu(ii): an efficient magnetically recoverable green nanocatalyst for the preparation of acridine and quinazoline derivatives in aqueous media at room temperature†

**DOI:** 10.1039/d1ra01373d

**Published:** 2021-04-29

**Authors:** Milad Kazemnejadi, Mohammad Ali Nasseri, Safoora Sheikh, Zinat Rezazadeh, Seyyedeh Ameneh Alavi Gol

**Affiliations:** Department of Chemistry, Faculty of Sciences, University of Birjand P. O. Box 97175-615 Birjand Iran miladkazemnejad@yahoo.com; Institut für Organische Chemie, Universität Regensburg Universitätsstr. 31 93053 Regensburg Germany

## Abstract

Saponin, as a green and available phytochemical, was immobilized on the surface of magnetite nanoparticles then doped with Cu ions (Fe_3_O_4_@Sap/Cu(ii)) and used as an efficient nanocatalyst for the synthesis of quinazoline and acridine derivatives, due to their high application and importance in various fields of science. Different spectroscopic and microscopic techniques were used for the catalyst characterization such as FT-IR, XRD, FE-SEM, EDX, TEM, TGA, VSM, BET, DLS, CV, and XPS analyses. All characterization data were correlated with each other so that the structure of the catalyst was accurately characterized. The reactions were performed in the presence of a low amount of Fe_3_O_4_@Sap/Cu(ii) (0.42 mol%) as a green catalyst in water over a short period of time. The results show well the effective role of saponin in solving the problem of mass transfer in aqueous medium, which is the challenge of many organic reactions in aqueous medium and in the presence of heterogeneous medium. High catalytic activity was found for the catalyst and high to excellent efficiency was obtained for all quinazoline (68–94% yield) and acridine (66–97% yield) derivatives in short reaction times (less than 1 hour) under mild reaction conditions in the absence of any hazardous or expensive materials. There is not any noticeable by-product found whether for acridine or quinazoline derivatives, which reflects the high selectivity. Two reasonable mechanisms were proposed for the reactions based on observations from control experiments as well as literature reports. The catalyst could be easily recovered magnetically for at least six consecutive runs with insignificant reactivity loss.

## Abstract 2

A highly efficient, robust, and green protocol has been developed for the synthesis of acridine and quinazoline derivatives in water under mild reaction conditions using a Fe_3_O_4_@Sap/Cu(ii) nanocomposite as an efficient heterogeneous catalyst.

## Graphical abstract

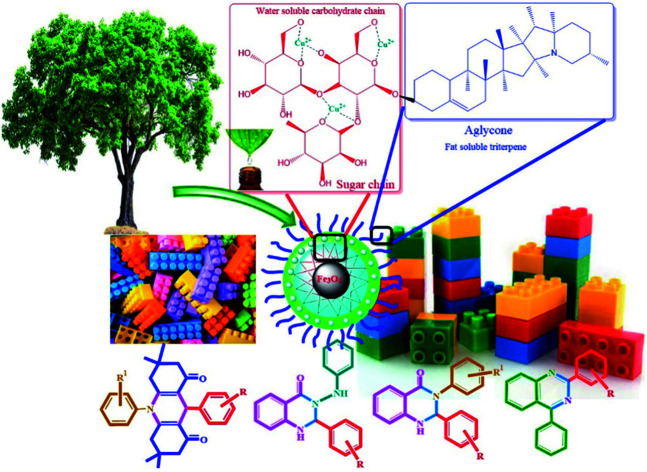

## 1. Introduction

In the context of sustainable eco-environmental aspects and saving energy,^1,2^ the production cycle of chemical industries is facing various constraints. Therefore, according to the existing needs during the past decade, the design and development of environmentally friendly recyclable organo-catalysts with desirable structural diversity and high selectivity, has become a big challenge for researchers and scientists.^3–5^ Hitherto, innovative catalysts have been developed as a fruitful strategy with the aid of natural organic compounds.^3,6,7^

**Fig. 1:**
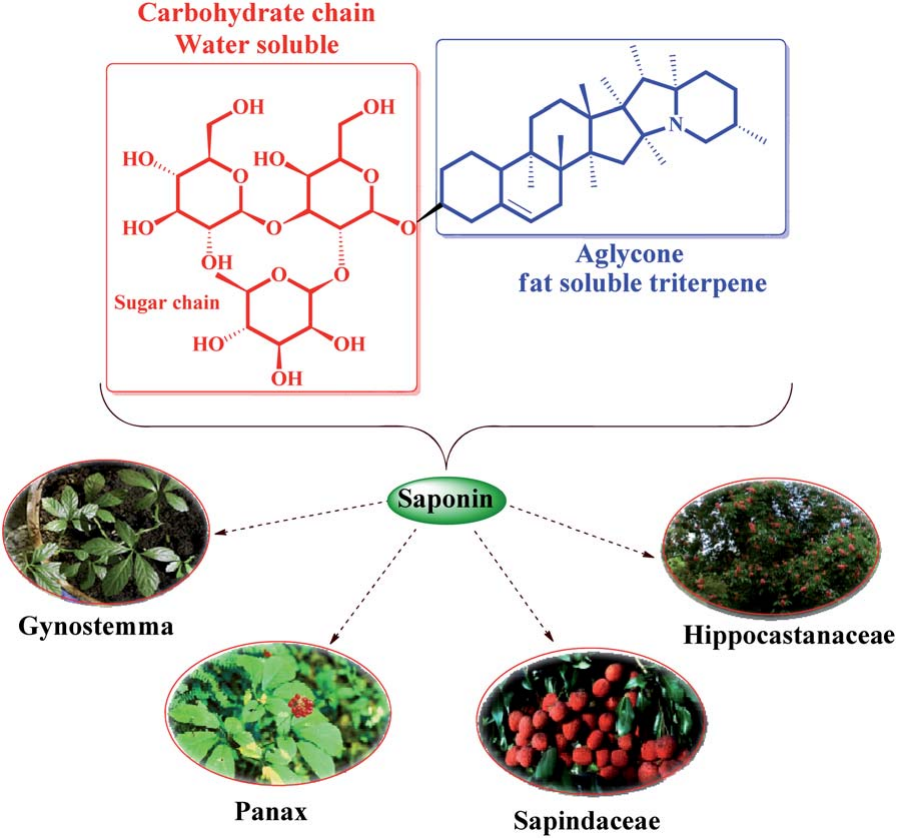
Several plants containing saponin chemical structure.

Saponins are a diverse group of plant amphipathic glycosides^8^ consisting of steroid or triterpenoid aglycone attached to one or more sugar chains.^9^ The saponins structure can be seen in many plants; such as Sapindaceae,^10^ Panax,^11^ Gynostemma,^12^ and Hippocastanaceae^13^ (Fig. 1). Use of the saponin is a suitable choice in the catalytic processes as a natural compound with the advantages of cheapness, availability, and interesting property arising from water- and fat-soluble parts.^14^ Also, considering the unique structure of saponins' with multiple saccharide chain structure and hydroxyl groups (as the hydrophilic moieties)^15^ and because of the wide variety of polycyclic structures (as the lipophilic moieties),^16^ they are widely used in phase-transfer catalytic processes and can be transferred between water and organic phases, easily.^17^ So, in order to synthesis a recoverable and efficient catalyst bearing transition metal complex^18^ using saponin, the immobilization of saponin as a green shell on Fe_3_O_4_ NPs, as a magnetic solid support, seems to be a smart strategy.

Acridines and quinazolines with a nitrogen-containing six-membered ring, are common in classes of nature and plant tissues.^19,20^ Acridines and quinazolines have various biological activities and pharmacophoric useful properties,^21^ such as antitumor properties,^22^ anti-cancer activity,^23^ anti-malarial activity,^24^ antiasthmatic,^25^ anti-allergic,^26^ and antiplatelet activity.^27^ Also, they play an important role as well in monitoring polymerization as a fluorescent molecular probe.^28,29^ In addition, their application as the n-type semiconductors in electro-luminescence devices have been also known.^30^ Therefore, due to the broad application of acridine and quinazoline derivatives, their synthesis is very important in synthetic organic chemistry.^31^

In the last few decades, a variety of synthetic methodologies have been expanded for the preparation of acridine and quinazoline derivatives.^32^ In this regard, it can be a point to the reactions such as domino synthetic protocols,^33^ aza-Diels–Alder reactions,^34^ oxidative cyclization,^35^ cyclo condensation reactions, and Bernthsen synthesis.^36^ Nevertheless, for researchers and manufacturers, from the point of view of material and energy saving, use of the inexpensive and more available materials and extending methodologies that rely on a green protocol, are desirable.^37^ Since, compared to complex processes, a one-pot synthesis in chemistry is an important strategy for advancing chemical reactions.^38^ Due to the above-mentioned advantages, the one-pot three-component condensation reaction between aromatic aldehyde, aniline, and dimedone for the synthesis of acridine is very common and noteworthy by chemists.^39^ In addition, one-pot three-component condensation reaction of (a) aromatic aldehydes, phenylhydrazine, and isatoic anhydride or (b) aromatic aldehydes, 2-aminobenzophenone, and ammonium acetate, for the synthesis of quinazolines are the common and key reactions in the synthesis of these compounds.^40^

To now, several methods have been reported for the one-pot multi-component condensation synthesis of acridine and quinazoline derivatives,^41^ employing a wide variety of catalysts such as Fe_3_O_4_/HT-SMTU-Zn^II^,^42^ SBA/AuNP,^43^ [Nbdm][OH],^44^ Co-aminobenzamid@Al-SBA-15,^45^ α-chymotrypsin,^46^ Pd(OAc)_2_,^47^ KCC-1/Pr-SO_3_H,^48^ and CuCl_2_,^49^ Co–alanine complex,^50^ magnetic praseodymium nanocatalyst,^51^ nano-Fe_3_O_4_-DOPA-SnO_2_,^52^ MNPs-NPB-SO_3_H,^53^ SDS^54^ and bentonite.^55^ Despite the usefulness of these catalysts, they also have some limitations including high toxicity, use of expensive materials, complex synthetic processes, long reaction times, and sometimes low yields.

Conventional catalytic processes using homogeneous catalysts are highly efficient. However, they show different impediments including the use of expensive or toxic catalytic systems, difficulty in separation, tedious work-up and waste discarding.^3^ In this point of view, the heterogeneous catalysts have emerged as a promising alternative, cover the most of defects of homogeneous catalysts such as reducing the waste production, providing a straightforward and simple separation and recovery of the catalysts. In this research, we have developed a new methodology for the synthesis of acridine and quinazoline derivatives, to minimize the above-mentioned limitations/drawbacks associated with previously reported methods. In this way, in accordance with green chemistry protocols, saponins as a cheap and green biomaterial were immobilized on magnetite NPs, then copper ions were coordinated to saponins (Fe_3_O_4_@Sap/Cu(ii)) as a phase-transfer, recoverable and reusable magnetic catalyst in organic synthesis. The ability to react in aqueous medium, due to the presence of saponin in the structure of the catalyst, not only solves the problem of mass transfer, but also facilitates the purification of products. This advantage is due to the presence of two hydrophilic and hydrophobic components in the saponin structure, which is a useful strategy for designing catalysts used in the aqueous phase to prepare organic compounds. Also, synergistic effect of Fe_3_O_4_ nanoparticles and saponin cause the increase the active surface of the catalyst, solid-phase stability and reduces agglomeration of magnetic nanoparticles, increase active sites for Cu retention capacity and so high activity of Fe_3_O_4_@Sap/Cu(ii) was expected to be desirable. In addition, the catalyst uses very cheap and available raw materials such as saponin, which along with water as a cheap and safe solvent, make the proposed method a cost-effective alternative to the previously proposed heterogeneous methods. The high stability of the catalyst along with compatibility with different types of organic substrates and the use of cheap, safe and available raw materials are among the other advantages of the methodology presented in this work.

The synthesis of acridine and quinazoline derivatives in the presence of Fe_3_O_4_@Sap/Cu(ii) was performed *via* a one-pot multicomponent cyclocondensation of aromatic aldehydes with (i) aromatic amine and dimedone, (ii) aromatic amine and isatoic anhydride, (iii) phenyl hydrazine and isatoic anhydride, and (iv) 2-aminobenzophenone and ammonium acetate (Scheme 5). All reactions were performed under mild conditions, *i.e.* water as a solvent at room temperature, with high to excellent yield. In addition, the catalyst was readily recycled for the six consecutive cycles for cyclocondensation reactions of the acridine and quinazoline, with no significant decrease in its activity.

## 2. Results and discussion

### 2.1. Catalyst characterization

**Fig. 2:**
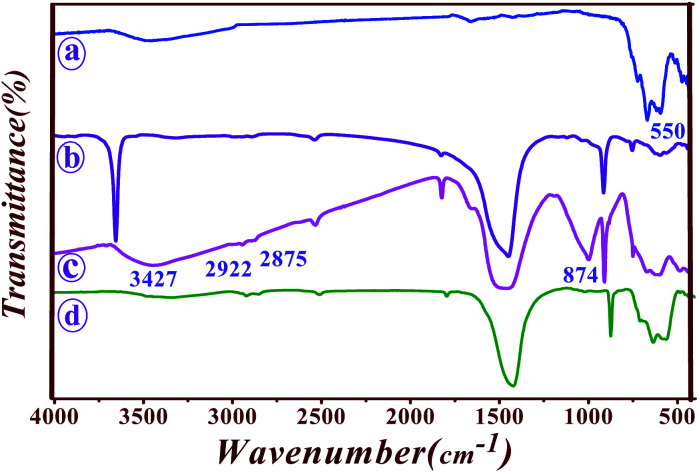
FT-IR spectra of (a) Fe_3_O_4_, (b) saponin, (c) Fe_3_O_4_@Sap, and (d) Fe_3_O_4_@Sap/Cu(ii).

FT-IR spectra of Fe_3_O_4_, saponin, Fe_3_O_4_@Sap, and Fe_3_O_4_@Sap/Cu(ii) compounds were shown in Fig. 2. The spectrum of Fe_3_O_4_ nanoparticles exhibited a strong absorption band at 559 cm^−1^ related to stretching Fe–O bond in Fe_3_O_4_ (Fig. 2a).^56^ The triterpenoid and glycoside sections of saponin demonstrated the absorptions of O–H, CH, C=O, C–O, and C=C bands at 3313, 2882, 890, and 1395 cm^−1^ in the saponin FT-IR spectrum (Fig. 2b).^57^ The spectrum of the immobilized saponin on iron oxide nanoparticles are shown with the eight characteristic peaks including Fe–O bond, =C–H vibrating o.o.p., C=C stretching of isomer *E*, C–O stretching, C=C stretching, C–H aliphatic, C–H aromatic, and OH stretching vibrations, that assigned respectively to 574, 713, 874, 1415, 1795, 2875, 2922, and 3427 cm^−1^ peaks (Fig. 2c).^57,58^ The absorption bands of Fe_3_O_4_@Sap/Cu(ii) were illustrated at 562 cm^−1^ (Fe–O bond), 635 cm^−1^ (=C–H vibrating o.o.p.), 874 cm^−1^ (C=C stretching of isomer *E*), 1427 cm^−1^ (C–O stretching), 1795 cm^−1^ (C=C stretching), 2857 cm^−1^ (C–H aliphatic), 2923 cm^−1^ (C–H aromatic), and 3342 cm^−1^ (OH stretching band), respectively (Fig. 2d).

**Fig. 3:**
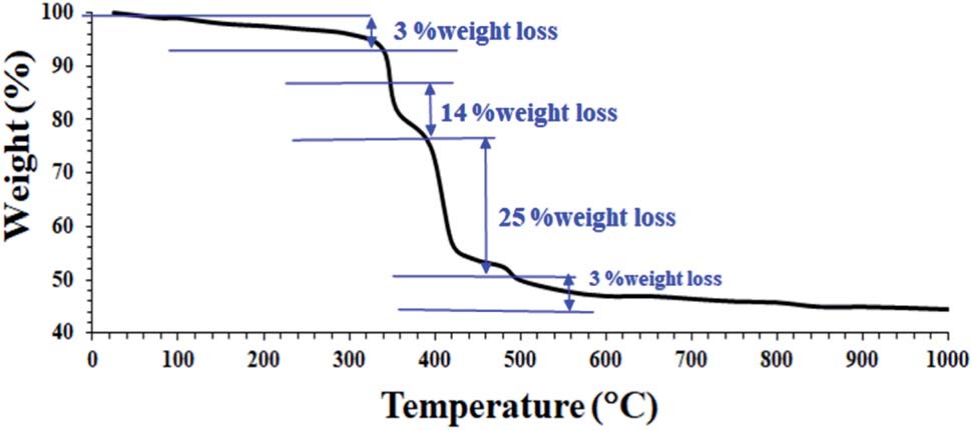
TGA curve of Fe_3_O_4_@Sap/Cu(ii) catalyst.

These results confirm the successful synthesis of the desired Fe_3_O_4_@Sap/Cu(ii). In order to better understand the thermal stability of the catalyst, the thermal behavior of Fe_3_O_4_@Sap/Cu(ii) was studied by TGA analysis (Fig. 3). TGA curve of the catalyst showed four weight loss steps totaling about 45%, which is consistent with the removal of adsorbed water (∼120 °C) on the surface, trapped water in the crystalline structure of the catalyst (∼220 °C), and the decomposition of fat chains of saponin structure immobilized on Fe_3_O_4_ NPs (∼350 °C).^59^ Drastically, the weight loss of about 25% was assigned to the decomposition of sugar chains of saponin. Oxidation of copper to Cu–O was responsible for the next weight loss (∼550 °C).^60^

Surface area and pore volume data of Fe_3_O_4_ and Fe_3_O_4_@Sap/Cu(ii) were studied by nitrogen adsorption/desorption isotherm analysis and the corresponding results were summarized in Table 1. The BET surface area of Fe_3_O_4_ was gradually decreased from 485 m^2^ g^−1^ to 459 m^2^ g^−1^, which is related to the loading of saponin/Cu on the magnetic surface. Therefore, following the obtained results from the BET analysis, the functionalization of Fe_3_O_4_ by saponin/Cu led to the raise of Fe_3_O_4_ nanoparticles pore size from 1.251 to 1.742 nm.

**Table 1:** Surface characteristic of Fe_3_O_4_ and Fe_3_O_4_@Sap/Cu(ii)

Entry	Sample	*S*_BET_ a (m^2^ g^−1^)	*V*_BET_ b (cm^3^ g^−1^)	*D*_P_ c (nm)
1	Fe_3_O_4_	485	0.806	1.251
2	Fe_3_O_4_@Sap/Cu(ii)	459	0.775	1.742

a BET specific surface area.

b Specific surface area.

c Pore volume.

**Fig. 4:**
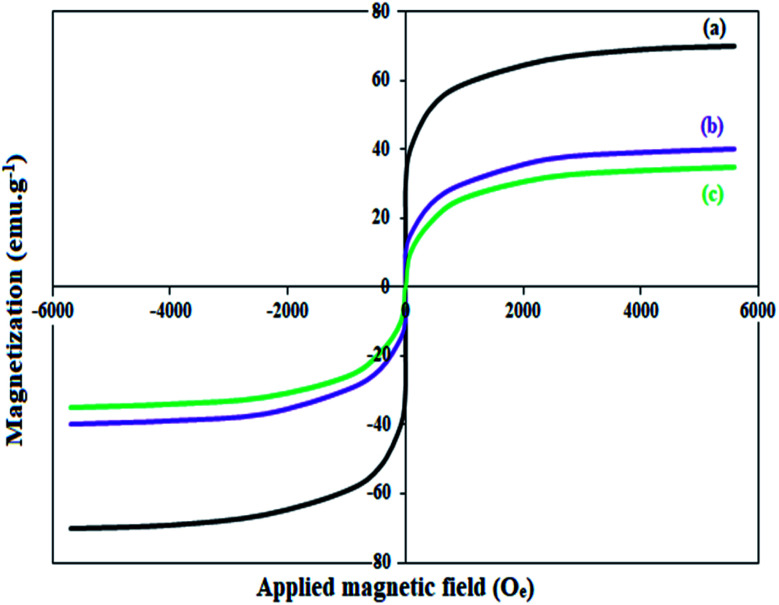
Magnetic behaviour of (a) Fe_3_O_4_, (b) Fe_3_O_4_@Sap, and (c) Fe_3_O_4_@Sap/Cu(ii).

The magnetic property of Fe_3_O_4_, Fe_3_O_4_@Sap, and Fe_3_O_4_@Sap/Cu(ii) was measured by VSM analysis at room temperature (Fig. 4). The VSM curves confirmed the superparamagnetic property for the all NPs.^61^ The magnetization values for Fe_3_O_4_, Fe_3_O_4_@Sap, and Fe_3_O_4_@Sap/Cu(ii) were 70, 40, and 35 emu g^−1^, respectively. Decrease of magnetization values of Fe_3_O_4_@Sap and Fe_3_O_4_@Sap/Cu(ii) exhibited the successful immobilization of saponin/Cu complex on the surface of Fe_3_O_4_ nanoparticles and acts as a diamagnetic barrier to the external magnetic field reaching the magnetic core. Despite this decrease, the catalyst was easily separated from the reaction medium by a simple external magnet in less than a minute. The amount of Fe, O, C, Cu, and N in the prepared Fe_3_O_4_@Sap/Cu(ii) were found to be 36.2, 33.0, 26.3, 3.0, and 1.5 wt%, respectively.

**Fig. 5:**
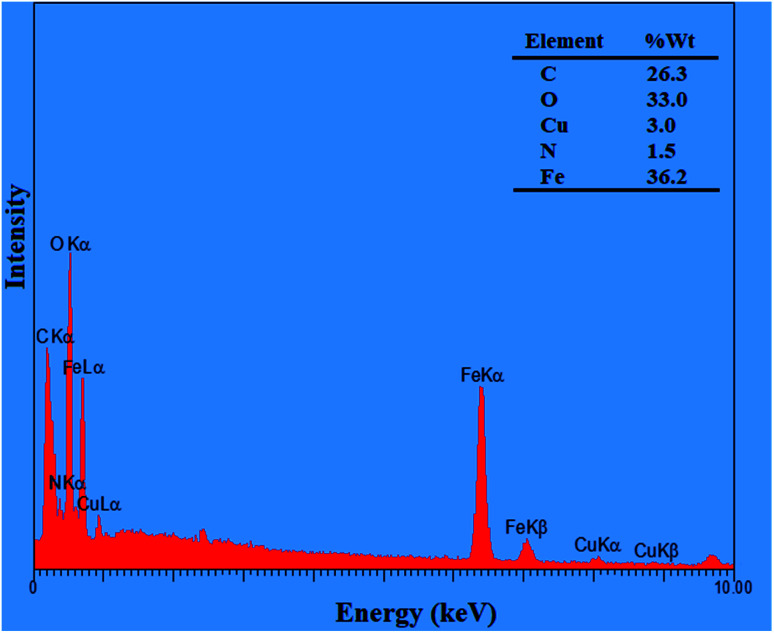
EDX analysis of Fe_3_O_4_@Sap/Cu(ii).

It worth noted that the amount of copper per gram of Fe_3_O_4_@Sap/Cu(ii) was 0.46 mmol, which was determined by the ICP analysis. The presence of Fe, O, C, Cu, and N elements were confirmed by EDX analysis, and there are no other elements, showing the purity of the sample (Fig. 5). In addition, EDX mapping analysis was also added to the ESI,† which shows the homogenous distribution of Fe, N, Cu, C and O (Fig. S1†).

**Fig. 6:**
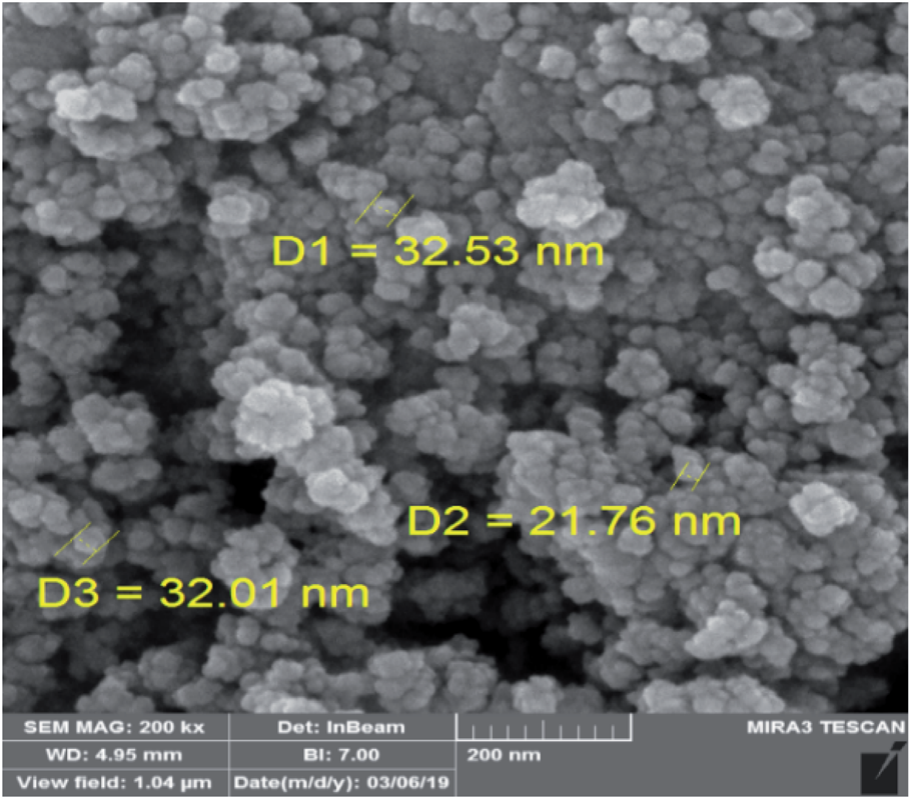
FE-SEM image of Fe_3_O_4_@Sap/Cu(ii) catalyst.

FE-SEM analysis shows the homogeneous spherical morphology and uniformity size for Fe_3_O_4_@Sap/Cu(ii) NPs (Fig. 6). Also, the FE-SEM image demonstrated the nano size of Fe_3_O_4_@Sap/Cu(ii).

**Fig. 7:**
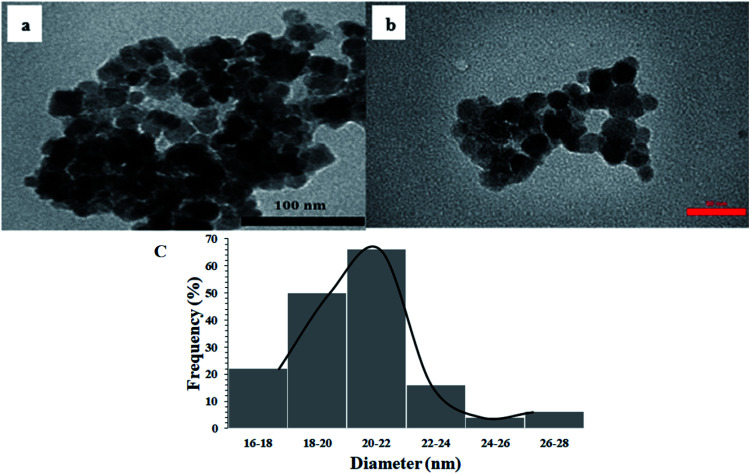
TEM image of (a) Fe_3_O_4_, (b) Fe_3_O_4_@Sap/Cu(ii) catalyst, and (c) size histogram of Fe_3_O_4_@Sap/Cu(ii) catalyst.

Fig. 7 shows the TEM images of Fe_3_O_4_ and Fe_3_O_4_@Sap/Cu(ii) catalyst. Spherical morphology with an identical size of particles of 20 nm was deduced from the images. Another interesting aspect related to TEM images was the complete dispersion of the catalyst in an ethanol medium, because of its hydrophilic section^62^ without any agglomeration (Fig. 7b). The size distribution histogram was estimated with a mean diameter of 20–22 nm for Fe_3_O_4_@Sap/Cu(ii) (Fig. 7c) in agreement with the TEM image.

**Fig. 8:**
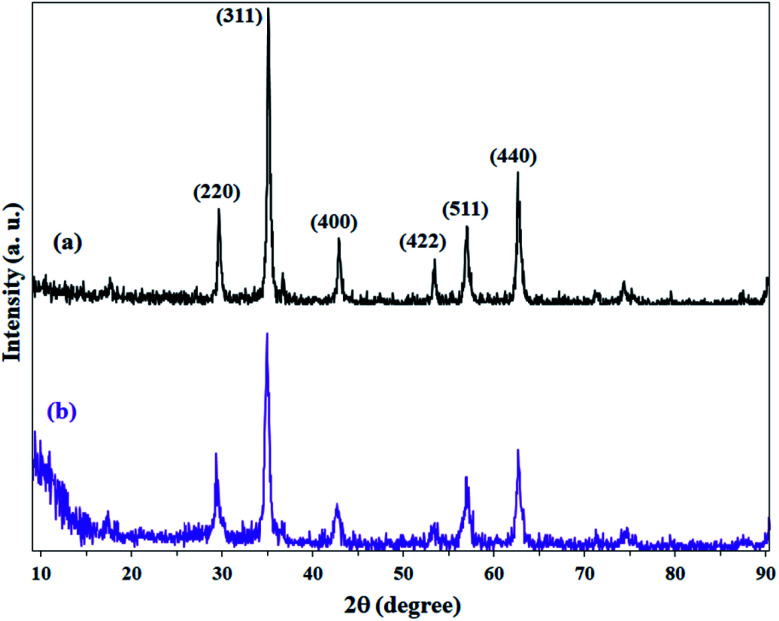
XRD patterns of (a) Fe_3_O_4_ and (b) Fe_3_O_4_@Sap/Cu(ii) catalyst.

XRD patterns of Fe_3_O_4_, Fe_3_O_4_@Sap/Cu(ii) nanostructures revealed six characteristic diffraction peaks at 2*θ* = 30.2°, 35.3°, 43.2°, 53.4°, 57.1° and 62.5° corresponding to (220), (311), (400), (422), (511), and (440) planes, which were completely in agreement with JCPDS card no. 19-629 for standard Fe_3_O_4_ (Fig. 8a). The diffraction patterns indicated obvious reduction peaks intensity after loading of saponin/Cu (1, 2) on the surface of Fe_3_O_4_ nanoparticles (Fig. 8b).

This decrease in intensity can also be directly attributed to the coating of nanoparticles by saponin/Cu having an amorphous structure, which is also a confirmation for the functionality of the Fe_3_O_4_ nanoparticles. Moreover, an amorphous peak at 2*θ* = 12° represents the amorphous structures immobilized on magnetite NPs and accordingly confirmed the successful functionalization of Fe_3_O_4_ NPs.

**Fig. 9:**
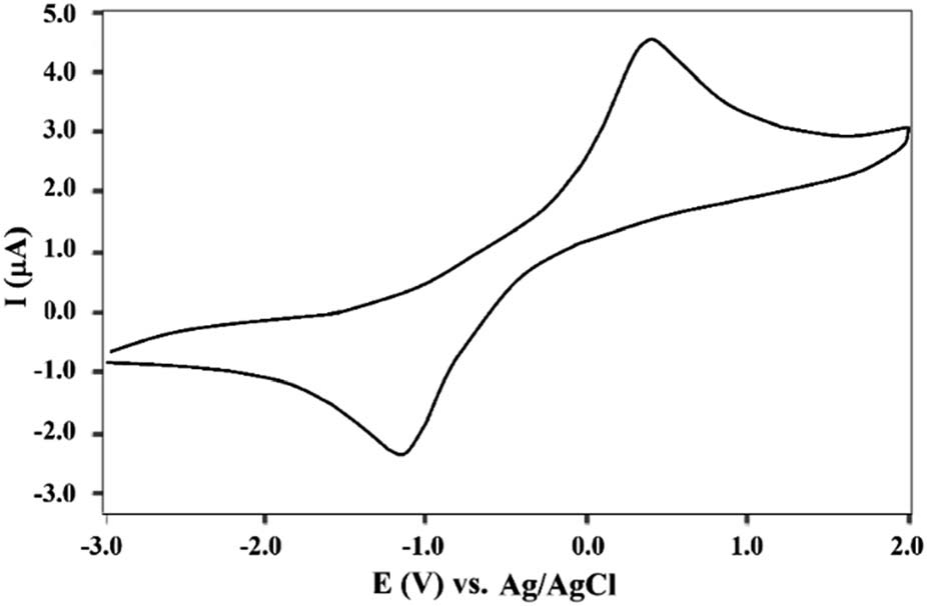
Cyclic voltammogram of Fe_3_O_4_@Sap/Cu(ii) in 0.1 M Britton–Robinson (BR) solution (pH = 7.0) with a scan rate of 100 mV s^−1^ at ambient temperature.

The electrochemical behavior of Fe_3_O_4_@Sap/Cu(ii) was investigated in the range of −3.0 to +2.0 V (Fig. 9). The resulting voltammogram shows the oxidation and reduction of copper sites with quasi-reversible behavior. A redox peak pair appearing at *E*_pc_ = +4.53 V and *E*_pa_ = −0.42 were assigned to the Cu(ii) → Cu(i) reduction and the Cu(i) → Cu(ii) oxidation respectively, confirming the certain redox-processes for the copper.^3,56,63^

**Fig. 10:**
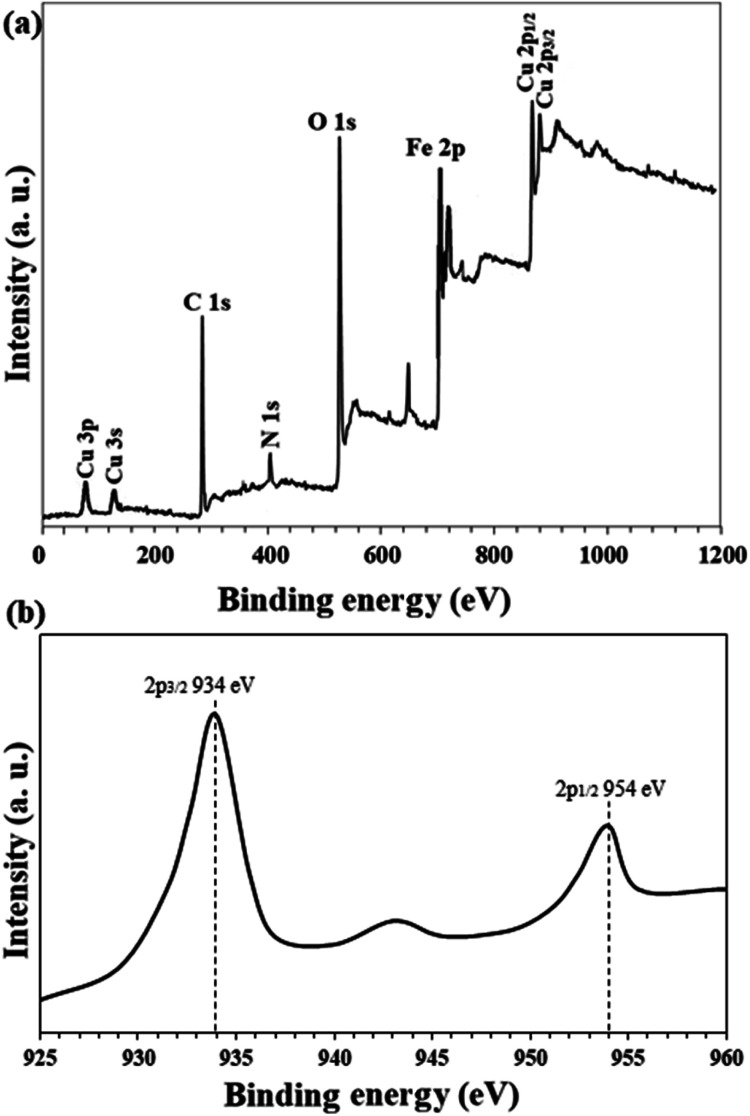
(a) Overall survey and (b) high resolution (normalized-energy corrected) Cu 2p XPS analysis of Fe_3_O_4_@Sap/Cu(ii) catalyst.

High resolution Cu 2p XPS analysis of Fe_3_O_4_@Sap/Cu(ii) catalyst in shown in Fig. 10. Two peaks at 934.0 eV (Cu 2p_3/2_) and 954 eV (Cu 2p_1/2_) represent the presence of Cu^2+^ (peak splitting = 20.0 eV) in the catalyst in agreement with the literature.^64^ Also, the spectrum shows a satellite at 943 eV, which is a characteristic for the Cu ions with the oxidation state of +2.^65^

### 2.2. Optimization of reaction parameters

**Fig. 11:**
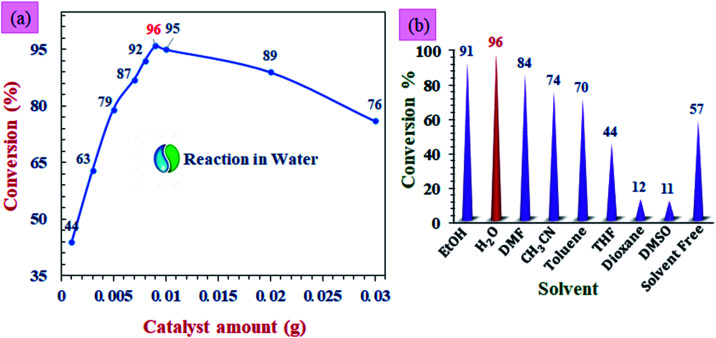
The screening of the (a) catalyst amount, and (b) solvent for the model reaction of benzaldehyde with dimedone and aniline. All the reactions were performed at room temperature.

The reaction parameters for the preparation of 1,8-dioxodecahydroacridines derivatives were investigated using Fe_3_O_4_@Sap/Cu(ii) nanoparticles as a catalyst. The reaction between benzaldehyde with dimedone and aniline was selected as a model reaction. For this goal, temperature, various solvents, and amount of catalyst parameters were investigated to determine the optimum conditions in the model reaction (Fig. 11).

As can be clearly seen in Fig. 11a the alteration of catalyst amount affecting the yield of product. The highest yield of product in the model reaction was obtained when 0.009 g (0.42 mol%) of the catalyst was used (96%). Also, without a catalyst, the reaction yield was trace; on the other hand, increment in the catalyst amount from 1 to 9 mg increases the yield from 44% to 96%, and also decreased the reaction time. The more increase in the amount of catalyst had no positive effect on the product yield, so that a further reduction in efficiency was also seen.^66^

Then, the effect of various solvents was investigated over the model reaction in the presence of the 0.42 mol% of Fe_3_O_4_@Sap/Cu(ii) at room temperature. The results in the presence of polar protic, polar aprotic, and non-polar solvents showed that in the aqueous medium in comparison with the other organic solvents, the reaction was more effective. These results were in agreement with the suggested catalyst structure bearing hydroxyl groups with the ability of phase transfer catalytic processes and transition between aqueous and organic phases. In Fig. 11b, it can be seen that the yield was greater in the presence of polar and protic solvents.^67^ The results showed that, in the water solvent, the reaction was more effective than other organic solvents (Fig. 11b).

### 2.3. Catalytic activity

The catalytic activity of Fe_3_O_4_@Sap/Cu(ii) was evaluated for the synthesis of quinazoline as well as acridine derivatives under optimal conditions (Tables 2 and 3). A variety of aldehydes bearing electron-withdrawing and electron-donating groups with several aniline derivatives were studied for the preparation of acridine and quinazoline derivatives. The results are summarized in Tables 2 and 3.

**Table 2:** One-pot synthesis of acridine derivatives in the presence of Fe_3_O_4_@Sap/Cu(ii) as a catalyst^a^

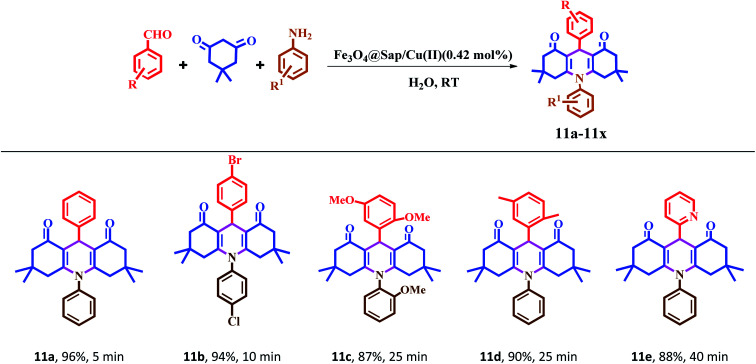
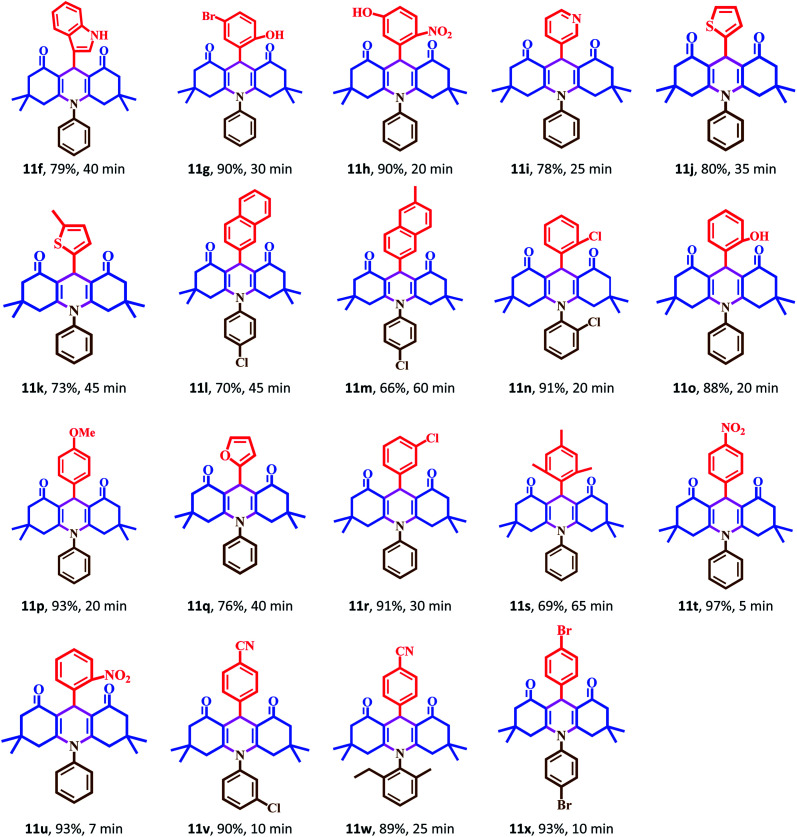

a Reaction conditions: aromatic aldehydes (1.0 mmol), aromatic amine (1.2 mmol), dimedone (2.0 mmol), Fe_3_O_4_@Sap/Cu(ii) (0.42 mol%), water (2.0 mL), room temperature; isolated yields%.

**Table 3:** One-pot synthesis of quinazoline derivatives in the presence of Fe_3_O_4_@Sap/Cu(ii) as a catalyst^a^

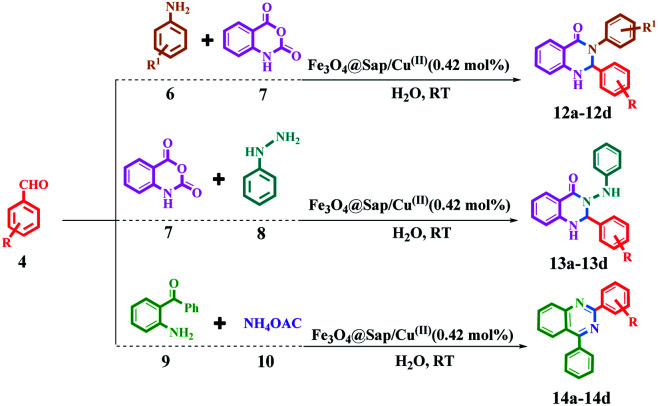
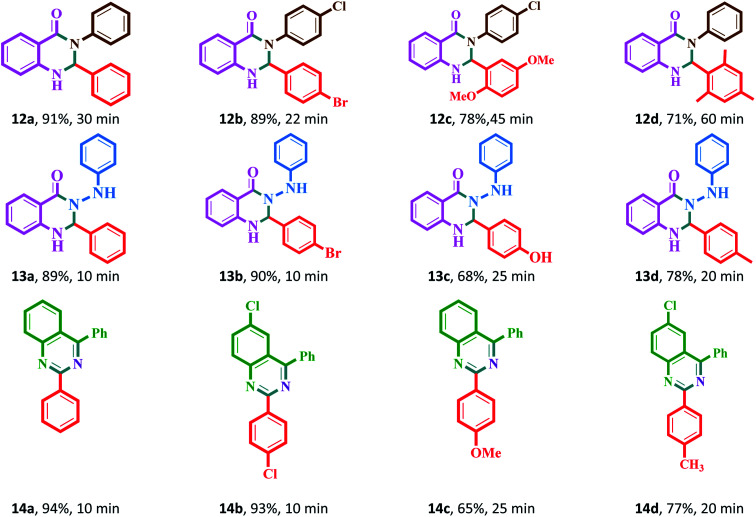

a Reaction conditions: aromatic aldehydes (1.0 mmol), aromatic amine (1.2 mmol) (or phenyl hydrazine (1 mmol) or ammonium acetate (1.5 mmol)), isatoic anhydride (1 mmol) or 2-aminobenzophenone (1 mmol), Fe_3_O_4_@Sap/Cu(ii) (0.42 mol%), water (2.0 mL), room temperature; isolated yields%.

It was notable that the both electron-releasing and electron-withdrawing groups, such as NO_2_, COOH, OCH_3_, and CH_3_, and halogens like Cl and Br properly react and produce high-efficiency products in a short reaction time. The aldehyde derivatives bearing electron-acceptor groups have greater efficiency in the reaction for the synthesis of acridines and quinazolines. The existence of stronger electron-donor groups on benzaldehyde delayed the reaction. For aldehydes bearing electron-acceptor groups, the positive center of the carbonyl group was more active and proceeds more easily nucleophilic attack and the time of reaction becoming shorter. Besides, heterocyclic aldehydes were also studied for the synthesis of acridine (Table 2). As is evident, products were obtained with good to excellent yields for both quinazoline and acridine derivatives in the presence of Fe_3_O_4_@Sap/Cu(ii) as a green catalyst in a short reaction time (Tables 2 and 3).

### 2.4. Mechanism study

#### 2.4.1. 1,8-Dioxo-decahydroacridine derivatives (11a–11x)

**Scheme 1:**
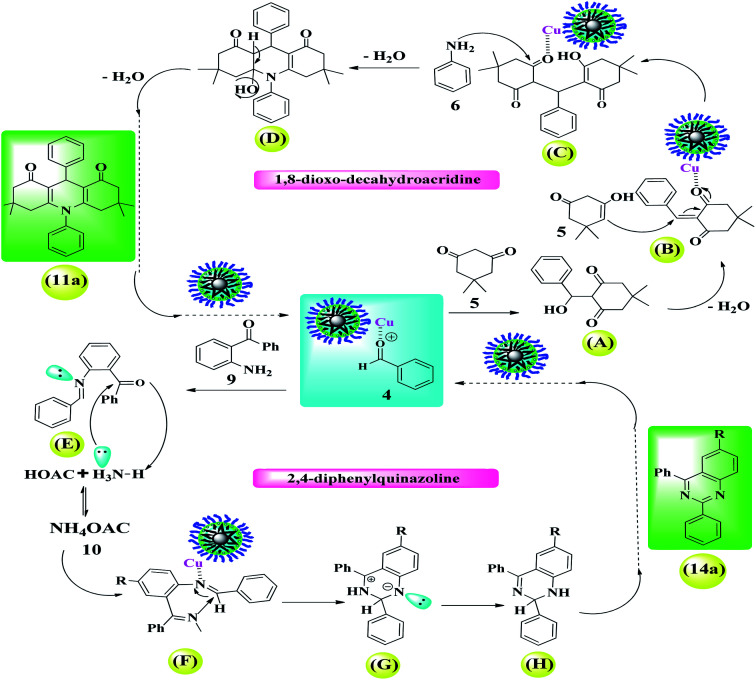
A plausible catalytic cycle for the preparation of 1,8-dioxo-decahydroacridine and 2,4-diphenylquinazoline derivatives catalyzed by Fe_3_O_4_@Sap/Cu(ii).

According to the mechanisms reported in the literature,^42^ a plausible reaction mechanism for the synthesis of 1,8-dioxo-dioxo-decahydroacridine derivatives in the presence of Fe_3_O_4_@Sap/Cu(ii) (3) has been suggested and shown in Scheme 1. The results showed that the presence of saponin on the catalyst surface causes the hydrophilic/hydrophobic feature and subsequently provides a suitable medium for the organic reactions in the aqueous medium. First, the forming hydrogen bonding between the acidic active sites in the presence of Fe_3_O_4_@Sap/Cu(ii) (3) and carbonyl groups in aromatic aldehyde (4) and dimedone (5), increases the electrophilic properties of carbonyl groups. Then, a condensation reaction of aromatic aldehyde (4) with dimedone (5) was formed and provide intermediate (A) (arylidene dimedone). By removal of a water molecule, intermediate (A) is converted to intermediate (B). Then, with a Michael addition of enolizable dimedone to intermediate (B), gives intermediate (C) in the presence of Fe_3_O_4_@Sap/Cu(ii) (3). In following, by a nucleophilic attack *via* the *N*-aniline (6) to the activated carbonyl group in intermediate C, intermediate (D) is produced. Subsequently, compound (D) with the removal of a water molecule is converted to the desired product (11a) (Scheme 1).

#### 2.4.2. 2,4-Diphenylquinazoline derivatives (14a–14d)

In Scheme 1, a plausible mechanism for the conversion of the aromatic aldehyde (4), 2-aminobenzophenone (9) and ammonium acetate (10) to 2,4-diphenylquinazoline (14a), in the presence of Fe_3_O_4_@Sap/Cu(ii) (3) as a green heterogeneous nanocatalyst was also proposed.^40^ In first, the reaction begins with the formation of a hydrogen bond between Fe_3_O_4_@Sap/Cu(ii) (3) and the carbonyl group of benzaldehyde (4). In the next step, a condensation reaction occurs between the activated benzaldehyde and 2-aminobenzophenone (9). Subsequently, the removal of a water molecule, aldimine intermediate (E) is formed. Then, the keto group of intermediate (E) reacts with the ammonium acetate, and forms the intermediate (F). In the next step, by forming a Cu–N bond, the imine group in intermediate (F) is activated by Fe_3_O_4_@Sap/Cu(ii) (14), and intermediate (G) is formed *via* a cyclization reaction. Then, with the transformation of the hydrogen in intermediate (G), compound (H) is produced. Finally, by aromatization of compound (H), the desired product (14a) is formed (Scheme 1).

#### 2.4.3. 2,3-Dihydroquinazolin-4(1*H*)-ones (12a–12d) and 2-phenyl-3-(phenylamino)-dihydroquinazoli-4(1*H*)-onesin (13a–13d)

**Scheme 2:**
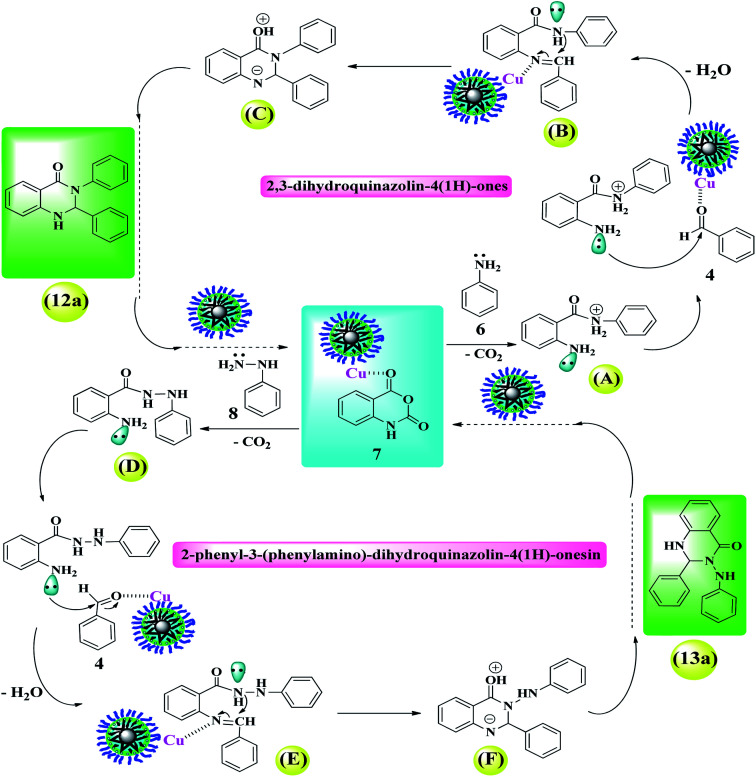
A plausible catalytic cycle for the preparation of 1,8-dioxo-decahydroacridine and 2,4-diphenylquinazoline derivatives catalyzed by Fe_3_O_4_@Sap/Cu(ii).

According to the obtained results in the literature,^48^ a plausible catalytic cycle for the preparation of 2,3-dihydroquinazolin-4(1*H*)-ones and 2-phenyl-3-(phenylamino)-dihydroquinazoli-4(1*H*)-onesin derivatives catalyzed by Fe_3_O_4_@Sap/Cu(ii) was proposed and shown in Scheme 2. At the first, the Cu centers as Lewis acidic sites in the Fe_3_O_4_@Sap/Cu(ii) (3), activates the carbonyl group of isatoic anhydride (7). In the next step, an *N*-nucleophilic attack takes place from the primary amine 6 (or 8) to the activated carbonyl, followed by the decarboxylation reaction, which was produced intermediate A (or D). Then, the amino group of intermediate A (or D) attacks to the activated benzaldehyde (4), and subsequently by removal of a water molecule, produces an imine intermediate B (or E). Then, the amide *via* an intermolecular nucleophilic attack on activated imine carbon in intermediate B (or E) led to the formation of intermediate C (or F). Finally, product 12a (13a) could be formed by a 1,5-proton shift of intermediate C (or F) (Scheme 2).

### 2.5. Control experiments

In order to prove the uniqueness of the catalytic activity of Fe_3_O_4_@Sap/Cu(ii), the model reaction was performed in the absence of catalyst, Cu(OAc)_2_, saponin, Fe_3_O_4_, Sap/Cu(ii), Fe_3_O_4_@Sap as a catalyst for the synthesis of 3,3,6,6-tetramethyl-9,10-diphenyl-3,4,6,7,9,10-hexahydroacridine-1,8(2*H*,5*H*)-dione (11a) (Table 4).

**Table 4:** Control experiments for the synthesis of 11aand 14a in water at room temperature

Entry	Cat. (0.009 g)	Time (min)	Yield (%)	
			11a^a^	14a^b^
1	Cu(OAc)_2_	60	10	10
2	Saponin	60	20	20
3	Fe_3_O_4_	60	35	35
4	Fe_3_O_4_@Sap/Cu(ii)	5	96	94
5	Sap/Cu(ii)	5	80	77
6	Fe_3_O_4_@Sap	5	69	66

a Reaction conditions: benzaldehyde (1 mmol), aniline (1.2 mmol), dimedone (2 mmol), Fe_3_O_4_@Sap/Cu(ii) (0.42 mol%), water (2.0 mL), room temperature; isolated yields%.

b Reaction conditions: benzaldehyde (1 mmol), 2-aminobenzophenone (1 mmol), ammonium acetate (1.5 mmol), Fe_3_O_4_@Sap/Cu(ii) (0.42 mol%), water (2.0 mL), room temperature; isolated yields%.

According to the results from Table 4, the reaction in the presence of Cu(OAc)_2_ and saponin gives only 10% and 20% in an hour, respectively. By coordinating copper (as a Lewis acid) on saponin, a significant improvement was observed in the reaction efficiency (Table 4, entry 5). This demonstrates that all parts of the catalyst participate in the transformation. Also, controlling tests were done for 14a and the results were reported in Table 4.

The significant performance of the catalyst in the aqueous medium as well as addressing concerns such as the mass transfer of organic molecules in the aqueous medium can be explained by the saponin structure immobilized on the nanoparticles. According to the results of control experiments, the results of acridine and quinazoline derivatives preparation are completely consistent with the theoretical approaches that the presence of catalytically active sites on nanoparticles significantly increases the efficiency, which can be seen in practice in the results obtained in Tables 2 and 3.

**Scheme 3:**
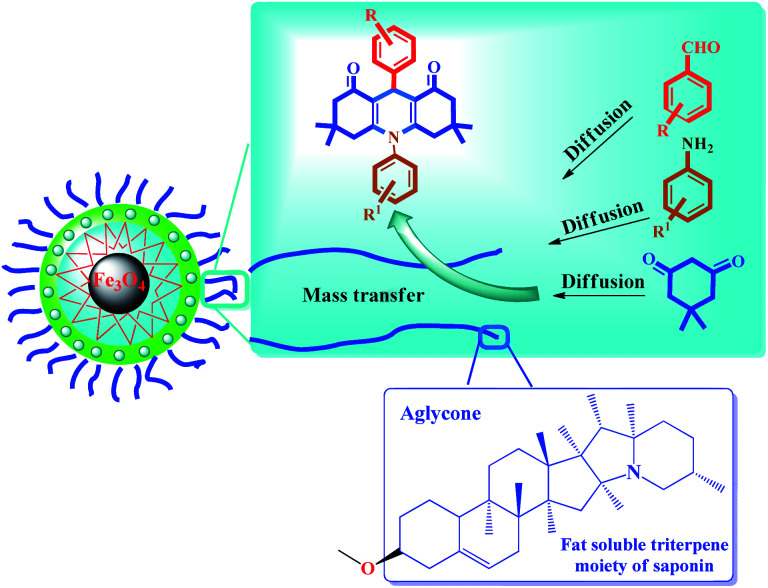
A simple and brief schematic view for the possible performance of Fe_3_O_4_@Sap/Cu(ii) in aqueous medium.

Scheme 3 shows the possible interaction of the catalyst and the reactants for the condensation reaction of benzaldehyde, aniline and dimedone (preparation of 11a) perfectly in accordance with the literature.^6,18,68^

The results were completely consistent with theoretical expectations, wherein the presence of lipophilic aglycone groups in the saponin structure causes the hydrophobic reactants to be directed (diffused) into the catalyst framework and (theoretically), consequently, the active catalytic centers (presence of copper groups) provides proper interaction between the surface of the catalyst and the raw materials causes them to condense and give the desired product (Scheme 3). Due to the insolubility of the product in the aqueous medium, the product is given out from the catalyst medium and acts as a driving force (as seen in Le Chatelier's principle). In agreement with the results of the control experiments, the reaction is often carried out by activated coordinated copper centers on the surface of the nanoparticles and saponins alone have very little catalytic activity to produce acridine and quinazoline compounds.

### 2.6. Recyclability

**Fig. 12:**
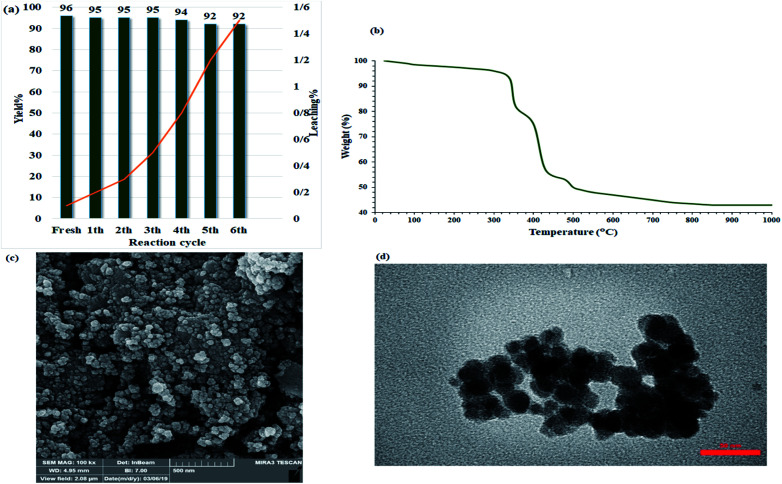
Recycling studies of Fe_3_O_4_@Sap/Cu(ii) in the acridine model reaction. (a) TGA curve, (b) FE-SEM image (c), and (d) TEM image of 6^th^ recovered Fe_3_O_4_@Sap/Cu(ii) catalyst.

In the heterogeneous catalysts field, catalyst recovering and recusing are important matters for environmental and practical considerations. In this way, the recyclability of the catalyst was studied over the model acridine reaction (between benzaldehyde, 5,5-dimethyl-1,3-cyclohexanedione, and aniline) at room temperature in water. As shown in Fig. 9a, the catalyst could be recovered and reused for at least six consecutive times without any notable reduction in catalytic activity (Fig. 9a). Fig. 12 shows that every recovery yields a very low-efficiency drop so that after the sixth cycles, the efficiency reached to 92% which is insignificant. In addition, the residual solution after each cycle was studied by ICP analysis to measure copper metal leaching. The presence of carbohydrate chains in the catalyst prevented metal leaching in aqueous media which remains catalytic activity for practical destinations due to its water stability. To investigate the stability and structure of the recovered catalyst (after 6th run), it was characterized by TGA, FE-SEM, and TEM analyses. The TGA curve of the recovered catalyst was quite similar to that of the fresh one (Fig. 12b), indicating that the catalyst retained its structure after repeated recovery and reuse, thus demonstrating its stability. In addition, the FE-SEM and TEM images of the recovered catalyst also showed the homogeneous and spherical morphology of the nanoparticles as same as the fresh one. More importantly, no significant agglomeration was seen in the images, reflecting the high dispensability of the nanoparticles (Fig. 12c and d). Therefore, the presence of hydrophilic section in the catalyst with higher ability to water absorption, causes to the better dispersion of the catalyst in the water solvent and subsequently provides a suitable FE-SEM and TEM images from the recovered catalyst.

### 2.7. Comparison with previously reported data

Finally, the characteristic of Fe_3_O_4_@Sap/Cu(ii) in the synthesis of acridine and quinazoline derivatives was compared to the recent reports. As shown in Table 5, the catalyst was showed various advantages in comparison with all the mentioned catalysts including highly efficient, robust, green, facile, and inexpensive catalyst, which in the presence of Fe_3_O_4_@Sap/Cu(ii), the synthesis of the compound 11a was accomplished at 5 minutes. Also, the synthesis of compounds 12a, 13a, and 14a was performed less than an hour. Another advantage of this catalyst is no use of toxic solvent and mild conditions.

**Table 5:** Comparison of the present methodologies with other reported catalysts for the synthesis of 11a, 12a, 13a, and 14a

Entry	Product	Catalyst	*T* (°C)	Solvent	Time (min)	Yield (%)	Number of recycles	Ref.
1	11a	Fe_3_O_4_@Sap/Cu(ii) (0.42 mol%)	RT	H_2_O	5	96	6	This work
2		Fe_3_O_4_/HT-SMTU-Zn^II^ (4 mol%)^a^	70	—	20	95	6	42
3		Co–alanine complex (5 mol%)	Reflux	EtOH	60	96	—	50
4		MNPs-NPB-SO_3_H (0.006 g)^bb^	Reflux	EtOH/H_2_O	20	94	5	53
5		SDS (0.2 g)^c^	90	H_2_O	9 h	70	—	54
6		NiFe_2_O_4_@SiO_2_@H_14_[NaP_5_W_30_O_110_] (0.02 g)	120	—	20	92	4	69
7		Pt NPs@GO (8 mg)	75	DMF	50	94	5	70
8		Vitamin B_1_ (10% mmol)	100	H_2_O	25	79	—	71
9		Nano-TiO_2_ (10% mmol)	75	—	30	80	8	72
10	12a	Fe_3_O_4_@Sap/Cu(ii) (0.42 mol%)	RT	H_2_O	30	91	6	This work
11		Fe_3_O_4_/SBA-15 (40 mg)^d^	Reflux	EtOH	210	75	5	73
12	13a	Fe_3_O_4_@Sap/Cu(ii) (0.42 mol%)	RT	H_2_O	10	89	6	This work
13		SrFe_12_O_19_ MNPs (20 mg)	120	—	15	87	4	74
14	14a	Fe_3_O_4_@Sap/Cu(ii) (0.42 mol%)	RT	H_2_O	10	94	6	This work
15		TEATCA (5 mol%)^ee^	Reflux	EtOH/H_2_O	10	85	4	75

a HT-SMTU = *S*-methylisothiourea.

b NPB = *N*-propyl-benzoguanamine.

c SDS = sodium 1 dodecanesulfonic.

d SBA = silica mesoporous.

e TEATCA = triethanolammonium-2,2,2-trichloroacetate.

## 3. Conclusion

Briefly, a highly efficient, powerful, and green protocol was developed to synthesize acridine and quinazoline derivatives in water under mild reaction conditions using a copper–saponin complex that was immobilized on Fe_3_O_4_ nanoparticles (Fe_3_O_4_@Sap/Cu(ii)). The magnetically recoverable nanocatalyst was characterized by FT-IR, FE-SEM, XRD, EDX, TEM, CV, DLS, XPS, and TGA analyses. This catalyst/methodology has several advantages, such as eco-friendly, mild reaction condition, low cost, low metal leaching, and compatibility with a wide variety of substrates, high efficiency, and recyclability. The reactions were carried out by a green solvent and diverse precursors, low reaction times, and high efficiency (65–96%), and there were no by-products. The results have occurred in the evaluated structures regardless of the presence of electron-donating and electron-withdrawing groups. The effectiveness was caused by water-soluble carbohydrate chain and aglycone fat-soluble triterpene in saponin. The saponin presence provides a proper medium, which is long aglycone chains was addressed mass transfer associated with organic compounds. On the other hand, the carbohydrate section with oxygen-containing heterocycles, facilitates copper coordination. In comparison with the previously reported methods, these benefits and characteristics making this catalyst, a reliable alternative methodology for the efficient preparation of acridine and quinazoline derivatives. Finally, the catalyst without any remarkable reactivity loss was recovered from the reaction mixture and reused for at least six consecutive runs. Given the wide range of properties of the present catalyst, its catalytic activity in other organic investigations is being investigated in another research project, including coupling reactions.

## 4. Experimental

### 4.1. Material and instruments

All substances were purchased from Sigma and Fluka suppliers and used as received without further treatment. Thin layer chromatography (TLC) was performed for monitoring reaction progress. FT-IR spectra were obtained using a JASCO FT/IR 4600 spectrophotometer using KBr pellet. The ^1^H-NMR (300 MHz) and ^13^C-NMR (75 MHz) spectra were recorded on a Bruker Avance DPX-300 spectrometer in deuterated solvents (CDCl_3_ and DMSO-*d*_6_) and TMS as an internal standard. Field emission scanning electron microscopy images were achieved on an SEM FEI Quanta 200. Gas chromatography (GC) using a Shimadzu-14B gas chromatograph equipped with an HP-1 capillary column and N_2_ as a carrier gas. EDX analyses were performed using a FESEM, JEOL 7600F apparatus equipped with a spectrometer of energy dispersion of X-ray from Oxford instruments. TEM microscopic images were performed on a Philips EM208S microscope operated at 100 kV. TGA analysis of the samples were performed on a Q600 model from TA company made in USA under nitrogen atmosphere with a heating rate of 10 °C min^−1^, and in the temperature range of 25–1000 °C. VSM curve of the samples was analyzed on a Lake Shore Cryotronics 7407 at room temperature. ICP experiments were conducted using a VARIAN VISTA-PRO CCD simultaneous ICP-OES instrument. XRD patterns were obtained on a Rigaku SmartLab. Cyclic voltammetry (CV) behavior (Electrochemical measurements) of the samples were performed on a CHI 1210A electrochemical workstation (CH Instrument, China) with a three-electrode system consisting of a standard Ag/AgCl as a reference electrode, a platinum wire electrode and a modified glassy carbon with the catalyst^76^ as an auxiliary and working electrode, respectively. The experiments were performed under a neutral atmosphere in the potential range from −2.0 to 2.0 V after 300 s accumulation under stirring with a scan rate of 100 mV s^−1^. The cell temperature was prepared at 25.0 ± 0.1 °C by means of a HAAKE D8 recirculating bath. Elemental analyses were accomplished on a PerkinElmer-2004 instrument.

### 4.2. Synthesis of Fe_3_O_4_@Sap/Cu(ii)

**Scheme 4:**
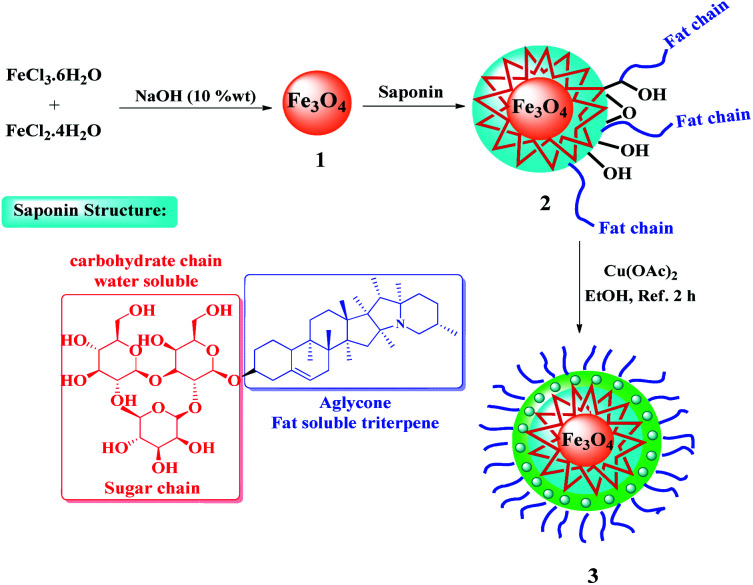
Preparation of Fe_3_O_4_@Sap/Cu(ii).

Fe_3_O_4_ NPs were prepared according to a previously described co-precipitation method.^5^ A mixture of Fe_3_O_4_ (1) nanoparticles (0.1 g) in H_2_O : EtOH (15 mL, 1 : 3 v/v) was sonicated at room temperature for 10 min. After that, saponin (0.2 g) was added to the above mixture and the reaction mixture was sonicated for 60 min. In the next step, NaOH 10% w/w (10 mL) was added dropwise to the solution underwent ultra-sonication for 30 min. The solution was stirred for 24 h. In the following, the obtained Fe_3_O_4_@Sap (2) nanoparticles were separated by an external magnet, washed with deionized water and dried under a vacuumed oven for 12 h. The coordination of copper ions to the Fe_3_O_4_@Sap (2) was performed with the addition of Fe_3_O_4_@Sap (1.0 g) to 50 mL of distilled water followed by the addition of 0.1 g Cu(OAc)_2_·H_2_O (0.1 g) to the reaction mixture. The reaction was refluxed with stirring for 4 h. Then, the product (3) (the catalyst) was filtered magnetically, washed with deionized water (3 × 10 mL), and then dried under vacuum (60 °C) for 24 h (Scheme 4).

### 4.3. General procedure for the synthesis of 1,8-dioxo-decahydroacridine catalyzed by Fe_3_O_4_@Sap/Cu(ii)

**Scheme 5:**
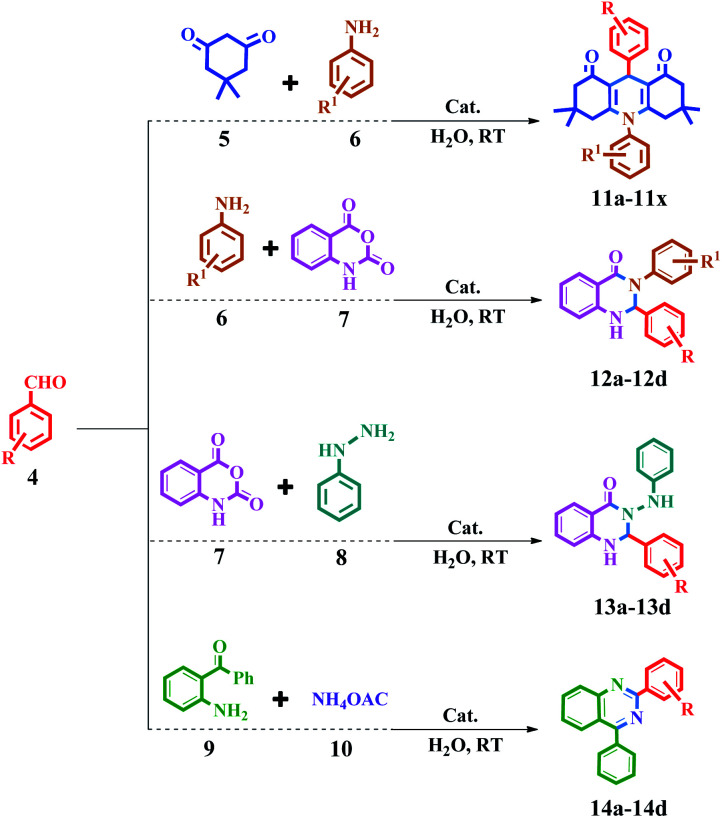
Synthesis of acridine and quinazoline derivatives in the presence of Fe_3_O_4_@Sap/Cu(ii) as a catalyst.

A mixture of aromatic aldehyde (1 mmol), 5,5-dimethyl-1,3-cyclohexanedione (2 mmol), aromatic amine (1.2 mmol) and Fe_3_O_4_@Sap/Cu(ii) (0.009 g, 0.42 mol%) in water (2 mL) was stirred at room temperature for an appropriate time (Scheme 5). The reaction progress was monitored by TLC continuously. After the completion of the reaction, ethyl acetate (5.0 mL) was added to the reaction mixture and the mixture was stirred for 5 min. Afterward, the catalyst was easily separated by applying an external magnetic field. In the next step, the reaction mixture was extracted three times with ethyl acetate (3 × 5 mL) and the combined organic phases were dried over Na_2_SO_4_. Ultimately, the product was obtained by removal of the ethyl acetate solvent under reduced pressure. The resulting crude product was purified by flash chromatography using ethyl acetate : *n*-hexane (2 : 1). The more results were summarized in Table 2. Yield: 66–97% (11a–11x).

### 4.4. General procedure for the synthesis of quinazoline derivatives catalyzed by Fe_3_O_4_@Sap/Cu(ii)

#### 4.4.1. General synthesis of 2,3-dihydroquinazolin-4(1*H*)-ones (12a–12d)

A mixture of aromatic aldehydes (1.0 mmol) with aromatic amine (1.2 mmol) and isatoic anhydride (1 mmol) and Fe_3_O_4_@Sap/Cu(ii) (0.42 mol%) in water (2.0 mL) was stirred at room temperature (Scheme 5). The progress of the reaction was monitored by TLC. The workup of this reaction was followed as the same as in Section 4.3. The more results were summarized in Table 3. Yield: 71–91% (12a–12d).

#### 4.4.2. General synthesis of 2-phenyl-3-(phenylamino)-dihydroquinazoli-4(1*H*)-onesin (13a–13d)

A mixture of aromatic aldehydes (1.0 mmol) with phenyl hydrazine (1 mmol) and isatoic anhydride (1 mmol) and Fe_3_O_4_@Sap/Cu(ii) (0.42 mol%) in water (2.0 mL) was stirred at room temperature (Scheme 5). The progress of the reaction was monitored by TLC. The workup of this reaction was followed as the same as in Section 4.3. The more results were summarized in Table 3. Yield: 68–90% (13a–13d).

#### 4.4.3. General synthesis of 2,4-diphenylquinazoline derivatives (14a–14d)

A mixture of aromatic aldehydes (1.0 mmol) with ammonium acetate (1.5 mmol), 2-aminobenzophenone (1 mmol) and Fe_3_O_4_@Sap/Cu(ii) (0.42 mol%) in water (2.0 mL) was stirred at room temperature (Scheme 5). The progress of the reaction was monitored by TLC. The workup of this reaction was followed as the same as in Section 4.3. The more results were summarized in Table 3.

## Conflicts of interest

There are no conflicts to declare.

## Data availability statement

The data that supports the findings of this study are available in the ESI† of this article.

## Supplementary Material

RA-011-D1RA01373D-s001
