# Supplementary material for: Fe3O4@Sap/Cu(ii): an efficient magnetically recoverable green nanocatalyst for the preparation of acridine and quinazoline derivatives in aqueous media at room temperature
Source: RSC Adv. 2021 Apr 29;11(26):15989–6003. doi: 10.1039/d1ra01373d (PMC9030076; doi:10.1039/d1ra01373d)
Supplement: RA-011-D1RA01373D-s001 [file RA-011-D1RA01373D-s001.pdf]

Electronic Supplementary Material (ESI) for RSC Advances.
This journal is © The Royal Society of Chemistry 2021

Supplementary Material

Fe_3O_4@Sap/Cu(II): An efficient magnetically recoverable green nanocatalyst for the preparation of acridine and quinazoline derivatives in aqueous media at room temperature

Milad Kazemnejadi,[a]* Mohammad Ali Nasseri[a]*, Safoora Sheikh,[a,b] Zinat Rezazadeh,[a] Seyyedeh Ameneh Alavi Gol[a]

[a] Department of Chemistry, Faculty of Sciences, University of Birjand, P. O. Box 97175-615, Birjand, Iran.

[b] Institut für Organische Chemie, Universität Regensburg, Universitätsstr. 31, 93053 Regensburg, Germany

Correspondence addressed: miladkazemnejad@yahoo.com; miladkazemnejadi@birjand.ac.ir;
Tel: +98-561-32202065.

Characterization data:

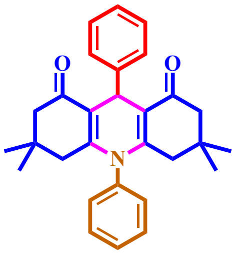

3,3,6,6-tetramethyl-9,10-diphenyl-3,4,6,7,9,10-hexahydroacridine-1,8(2H,5H) -dione (11a).

Yellow solid, m.p. 252 °C. ^{1}H NMR (300 MHz, $CDCl_3$): δ (ppm): 0.86 (s, 6H, $2CH_3$), 0.94 (s, 6H, $2CH_3$), 2.14 (d, 2H, J = 16.4 Hz,), 2.23 (d, 2H, J = 16.4 Hz,), 2.24–2.41 (m, 4H, $2CH_2$), 5.52 (s, 1H, CH), 7.01 (s, 1H, ArH), 7.04 (d, 2H, J = 6.2 Hz, ArH), 7.12– 7.28 (m, 5H, ArH), 7.34 (d, 2H, J = 6.2 Hz, ArH).^{13}C NMR (75 MHz, CDCl3): δ(ppm): 22.3, 23.8, 35.2, 42.4, 52.5, 125.2, 126.8, 127.5, 129.2, 131.5, 134.2, 136.9, 146.8, 148.3, 150.3, 166.2, 212.6.[1]

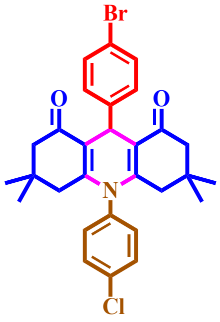

9-(4-bromophenyl)-10-(4-chlorophenyl)-3,3,6,6-tetramethyl-3,4,6,7,9,10-hexahydroacridine 1,8(2H,5H)-dione (11b).

White solid, m.p. 305 °C. ^{1}H NMR (300 MHz, $CDCl_3$): δ (ppm): 0.85 (s, 6H, $2CH_3$), 0.91 (s, 6H, $2CH_3$), 1.99 (d, 2H, J = 16.0 Hz, CH_2), 1.98 (d, 2H, J = 16.0 Hz, CH_2), 2.15-2.55 (m, 4H, $2CH_2$), 4.99 (s, 1H, CH), 7.17-7.44 (m, 4H, ArH), 7.49-7.52 (m, 4H, ArH).^{13}C NMR (75 MHz, $CDCl_3$): δ(ppm): 26.5, 29.9, 32.2, 32.5, 41. 5, 49.9, 113.1, 119.2, 130.3, 130.5, 131.2, 134.4, 137.6, 145.9, 150.8, 198.0.[1]

9-(2,5-dimethoxyphenyl)-10-(2-methoxyphenyl)-3,3,6,6-tetramethyl-3,4,6,7,9, 10-hexahydroacridine-1,8(2H,5H)-dione (11c).

White solid, m.p. 291 °C. ^{1}H NMR (300 MHz, $CDCl_3$): δ (ppm): 0.72 (s, 6H, $2CH_3$), 0.88 (s, 6H, $2CH_3$), 1.87 (dd, 4H, J = 17.4 Hz, J = 15.9 Hz, $2CH_2$), 2.24-2.16 (m, 4H, $2CH_2$), 3.70 (s, 6H, $2OCH_3$), 3.76 (s, 3H, OCH_3), 4.99 (s, 1H, CH), 7.22-7.01 (m, 4H, ArH), 7.65-7.40 (m, 3H, ArH), ^{13}C NMR (75 MHz, $CDCl_3$): δ(ppm): 26.5. 26.8, 29.7, 31.4, 31.7, 32.4, 50.0, 55.3, 113.7, 113.8, 128.9, 130.5,

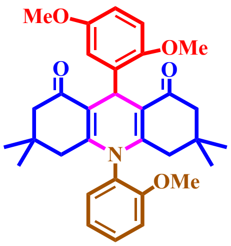

134.3, 137.8, 138.9, 150.2, 157.7, 197.5.[2]

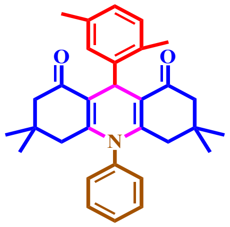

9-(2,5-dimethylphenyl)-3,3,6,6-tetramethyl-10-phenyl-3,4,6,7,9,10-hexahydroacridine-1,8(2H,5H)-dione (11d).

White solid, m.p. 307 °C. ^{1}H NMR (300 MHz, $CDCl_3$): δ (ppm): 0.72 (s, 6H, $2CH_3$), 0.88 (s, 6H, $2CH_3$), 1.76 (d, 2H, J = 15.9 Hz, CH_2), 2.01 (d, 2H, J = 15.9 Hz, CH_2), 2.25-2.17 (m, 4H, $2CH_2$), 3.37 (s, 6H, $2CH_3$), 5.05 (s, 1H, CH), 7.23-7.00 (m, 3H, ArH), 7.65-7.42 (m, 5H, ArH); ^{13}C NMR (75 MHz, $CDCl_3$): δ(ppm): 26.5, 27.2, 29.1, 31.8, 32.3, 32.4, 41.3, 50.0, 113.6, 127.8, 128.4, 128.9, 134.3, 135.1, 137.8, 143.6, 150.4, 195.5.[2]

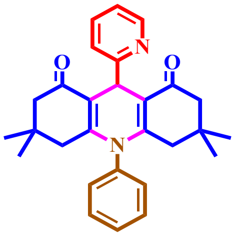

3,3,6,6-tetramethyl-10-phenyl-9-(pyridin-2-yl)-3,4,6,7,9,10-hexahydroacridine-1,8(2H,5H)-dione (11e).

Light brown solid, m.p. 334 °C. ^{1}H NMR (300 MHz, $CDCl_3$): δ (ppm): 0.88 (s, 6H, $2CH_3$), 0.93 (s, 6H, $2CH_3$), 1.88 (s, 4H, $2CH_2$), 2.12 (s, 4H, $2CH_2$), 4.98 (s, 1H, CH), 7.24–7.58 (m, 5H, ArH), 7.22-8.66 (m, 4H, ArH). ^{13}C NMR (75 MHz, $CDCl_3$): δ(ppm): 23.9, 24.9, 34.3, 42.5, 56.3, 129.9, 132.2, 140.9, 149.6, 151.9, 157.2, 167.3, 214.8.[2]

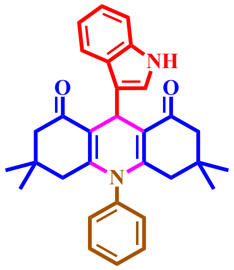

9-(1H-indol-3-yl)-3,3,6,6-tetramethyl-10-phenyl-3,4,6,7,9,10-hexahydroacridine-1,8(2H,5H)-dione (11f).

Light gray solid, m.p.> 350 °C. ^{1}H NMR (300 MHz, $CDCl_3$): δ (ppm): 1.00 (s, 6H, $2CH_3$), 1.17 (s, 6H, $2CH_3$), 1.88 (s, 4H, $2CH_2$), 1.99 (s, 4H, $2CH_2$), 5.15 (s, 1H, CH), 6.99 (s, 1H, CH Indole), 7.06-7.57 (m, 4H, Indole), 7.24-7.59 (m, 5H, ArH), 10.81 (d, 1H, NH). ^{13}C NMR (75 MHz, $CDCl_3$): δ(ppm): 27.8, 33.6, 43.2, 51.4, 61.2, 111.3, 120.6, 121.7, 127.5, 129.8, 132.6, 135.7, 136.2, 138.9, 144.2, 153.8, 153.9, 165.2, 209.[3]

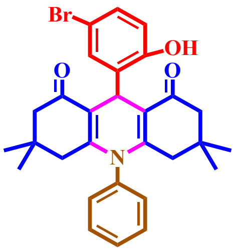

9-(5-bromo-2-hydroxyphenyl)-3,3,6,6-tetramethyl-10-phenyl-3,4,6,7,9,10 hexahydroacridine-1,8(2H,5H)-dione (11g).

White solid, m.p. 342 °C.^{1}H NMR (300 MHz, $CDCl_3$): δ (ppm): 0.64 (s, 6H, $2CH_3$), 0.74 (s, 6H, $2CH_3$), 1.59 (d, 2H, J = 17.1 Hz, CH_2) 1.84 (d, 2H, J = 15.8 Hz, CH_2), 1.94-1.96 (m, 4H, $2CH_2$), 4.94 (s, 1H, CH), 6.65-6.76 (m, 3H, ArH), 7.01-7.42 (m, 5H, ArH), 10.14 (s, 1H, OH). ^{13}C NMR (75 MHz, $CDCl_3$): δ(ppm): 27.5, 28.8, 31.4, 32.9, 40.9, 51.6, 113.1, 116.3, 118.9, 131.3, 131.6, 131.9, 132.2, 138.9, 147.4, 154.8, 108.6.[1]

9-(5-hydroxy-2-nitrophenyl)-3,3,6,6-tetramethyl-10-phenyl-3,4,6,7,9,10-hexahydroacridine-1,8(2H,5H)-dione (11h).

Yellow solid, m.p. 292 °C. ^{1}H NMR (300 MHz, $CDCl_3$): δ (ppm): 0.78 (s, 6H, $2CH_3$), 0.90 (s, 6H, $2CH_3$), 1.74 (d, 2H, J = 16.5 Hz, CH_2), 2.00 (d, 2H, J = 7.0 Hz, CH_2), 2.11-2.14 (m, 4H, $2CH_2$), 5.19 (s, 1H, CH), 6.55 (d, 1H, J = 8.00 Hz, ArH), 6.79 (d, 1H, J = 7.8 Hz, ArH), 7.02 (d, 2H, J = 8.8 Hz, ArH), 7.17 (s, 2H,

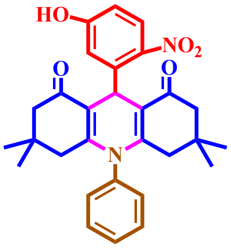

ArH), 7.39 (d, 1H, J = 7.6 Hz, ArH), 7.61 (d, 1H, J = 7.9 Hz, ArH), 10.44 (s, 1H, OH). ^{13}C NMR (75 MHz, $CDCl_3$): δ(ppm): 26.9, 29.1, 30.6, 31.8, 41.2, 53.6, 114.4, 116.8, 119.4, 131.9, 132.2, 132.9, 133.8, 137.9, 140.9, 164.7, 210.3.[3]

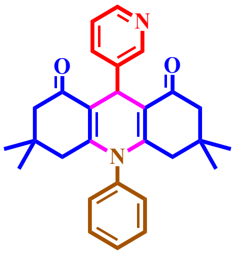

3,3,6,6-tetramethyl-10-phenyl-9-(pyridin-3-yl)-3,4,6,7,9,10-hexahydroacridine-1,8(2H,5H)-dione (11i).

Light brown solid, m.p. 321 °C. ^{1}H NMR (300 MHz, $CDCl_3$): δ (ppm): 0.72 (s, 6H, $2CH_3$), 0.88 (s, 6H, 2CH3), 1.70 (d, 2H, J =18 Hz, CH_2), 1.99 (d, 2H, J = 18 Hz, CH_2), 2.25-2.18 (m, 4H, $2CH_2$), 5.04 (s, 1H, CH), 7.10 (t, 1H, J = 6 Hz, ArH), 7.27-7.22 (m, 2H, ArH), 7.40-7.37 (m, 2H, ArH), 7.66- 7.62 (m, 2H, Pyridine), 8.13 (td, 1H, J = 7.8 Hz, J = 1.8 Hz, Pyridine), 8.76 (s, 1H, Pyridine), ^{13}C NMR (75 MHz, $CDCl_3$): δ(ppm): 21.6, 29.6, 32.5, 32.6, 40.8, 50.9, 113.6, 125.5, 126.7, 128.5, 140.1, 147.1, 149.5, 150.6, 151.3, 195.5.[2]

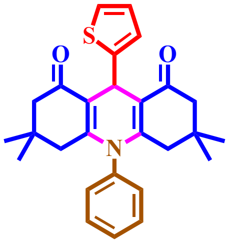

3,3,6,6-tetramethyl-10-phenyl-9-(thiophen-2-yl)-3,4,6,7,9,10-hexahydroacridine-1,8(2H,5H)-dione (11j).

Green light solid, m.p. 198 °C. ^{1}H NMR (300 MHz, $CDCl_3$): δ (ppm): 0.86 (s, 6H, $2CH_3$), 1.01 (s, 6H, $2CH_3$), 1.91-2.30 (m, 8H, $4CH_2$), 4.69 (s, 1H, CH), 7.23-7.46 (m, 3H, Thiophene), 7.28-7.56 (m, 5H, Ar-H). ^{13}C NMR (75 MHz, $CDCl_3$): δ(ppm): 27.9, 33.9, 37.4, 43.3, 53.7, 58.7, 117.5, 120.6, 124.0, 127.0, 129.0, 132.3, 133.7, 159.6, 194.9.[4]

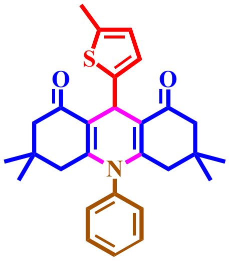

3,3,6,6-tetramethyl-9-(5-methylthiophen-2-yl)-10-phenyl-3,4,6,7,9,10-hexahydroacridine-1,8(2H,5H)-dione (11k).

Green light solid, m.p. 222 °C. ^{1}H NMR (300 MHz, $CDCl_3$): δ (ppm): 1.28 (s, 6H, $2CH_3$), 1.31 (s, 6H, $2CH_3$), 2.44 (s, 4H, $2CH_2$), 2.72 (s, 4H, $2CH_2$), 2.98 (s, 3H, CH_3), 4.65 (s, 1H, CH), 6.23 (d, 1H, Thiophene), 6.77 (d, 1H, Thiophene), 7.21-7.66 (m, 5H, Ar-H). ^{13}C NMR (75 MHz, $CDCl_3$): δ(ppm): 16.6, 21.9, 22.7, 31.2, 43.4, 55.1, 124.6, 126.8, 129.8, 132.2, 135.9, 137.8, 148.4, 152.6, 154.1, 164.2, 202.4.[4]

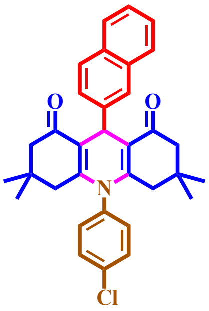

10-(4-chlorophenyl)-3,3,6,6-tetramethyl-9-(naphthalen-2-yl)-3,4,6,7,9,10-hexahydroacridine-1,8(2H,5H)-dione (11l).

Pale pink solid, m.p.> 350 °C. ^{1}H NMR (300 MHz, $CDCl_3$): δ (ppm): 0.84 (s, 6H, $2CH_3$), 0.96 (s, 6H, $2CH_3$), 2.05-2.14 (s, 4H, $2CH_2$), 2.20-2.26 (s, 4H, $2CH_2$), 5.27 (s, 1H, CH), 7.09 (d, 2H, J= 6.0 Hz, Ar-H), 7.25 (d, 2H, J= 8.0 Hz, Ar-H), 7.36-7.46 (m, 3H, Ar-H), 7.55 (d, 2H, J= 6.5 Hz, Ar-H), 7.69 (2H, d, J= 8.3 Hz, Ar-H). ^{13}C NMR (75 MHz, $CDCl_3$): δ(ppm): 27.1, 28.5, 30.7, 31.9, 40.2, 125.6, 126.4, 126.9, 127.6, 128.2, 128.4, 128.6, 129.5, 133.8, 137.7, 145.5, 145.9, 164.8, 208.6.[5]

10-(4-chlorophenyl)-3,3,6,6-tetramethyl-9-(6-methylnaphthalen-2-yl)-3,4,6,7,9,10-hexahydroacridine-1,8(2H,5H)-dione (11m).

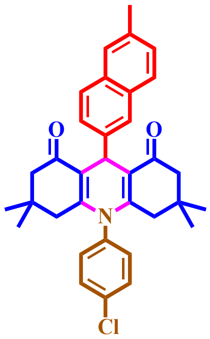

Pale pink solid, m.p.> 350 °C. ^{1}H NMR (300 MHz, $CDCl_3$): δ (ppm): 1.08 (s, 6H, $2CH_3$), 1.21 (s, 6H, $2CH_3$), 2.19 (s, 4H, $2CH_2$), 2.69 (s, 3H, CH_3), 2.71 (s, 4H, $2CH_2$), 5.27 (s, 1H, CH), 7.64-7.98 (m, 6H, Ar-H), 7.72-7.88 (m, 4H, Ar-H). ^{13}C NMR (75 MHz, $CDCl_3$): δ(ppm): 13.3, 23.5, 23.96, 33.3, 61.5, 124.8, 125.9, 128.0, 128.3, 128.3, 129.0, 129.6, 130.3, 130.7, 132.2, 133.0, 133.8, 133.9, 136.6, 144.5, 145.6, 154.4, 199.9.[5]

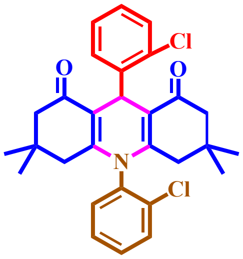

9,10-bis(2-chlorophenyl)-3,3,6,6-tetramethyl-3,4,6,7,9,10-hexahydroacridine-1,8(2H,5H)-dione (11n).

Yellow solid, m.p. 272 °C. ^{1}H NMR (300 MHz, $CDCl_3$): δ (ppm): 0.72 (s, 6H, $2CH_3$), 0.88 (s, 6H, $2CH_3$), 1.78 (d, 2H, J = 17.43 Hz, CH_2), 2.00 (d, 2H, J = 16.00 Hz, CH_2), 2.16-2.22 (m, 4H, $2CH_2$), 5.05 (s, 1H, CH), 7.21-7.52 (m, 4H, Ar-H), 7.48 (d, 2H, J = 5.44 Hz, 10.58, Ar-H), 7.68 (d, 2H, J = 8.84 Hz, Ar-H). ^{13}C NMR (75 MHz, $CDCl_3$): δ(ppm): 26.0, 29.7, 32.3, 32. 4, 41.3, 50.0, 113.5, 126.6, 127.7, 128.3, 130.5, 134.3, 137.7, 146.5, 150.5, 198.5.[6]

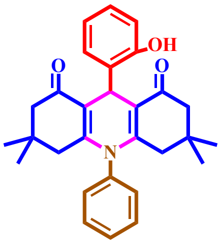

9-(2-hydroxyphenyl)-3,3,6,6-tetramethyl-10-phenyl-3,4,6,7,9,10-hexahydroacridine-1,8(2H,5H)-dione (11o).

Yellow solid, m.p. 247 °C. ^{1}H NMR (300 MHz, $CDCl_3$): δ (ppm): 0.82 (s, 6H, $2CH_3$), 0.92 (s, 6H, $2CH_3$), 1.48 (d, J = 15.4 Hz, 2H, CH_2), 2.02 (d, J = 15.4, 2H, CH_2), 2.28–248 (m, 4H, $2CH_2$), 5.48 (s, 1H, CH), 7.12 (d, J = 6.8 Hz, 2H, ArH), 7.18–7.23 (m, 4H, ArH), 7.36 (d, J = 6.4 Hz 2H, ArH), 9.11 (s, 1H, OH). ^{13}C NMR (75 MHz, $CDCl_3$): δ(ppm): 22.8, 23.6, 33.2, 41.2, 55.2, 123.2, 127.8, 129.2, 131.9, 136.2, 138.9, 148.4, 152.7, 153.5, 165.2, 212.6.[6]

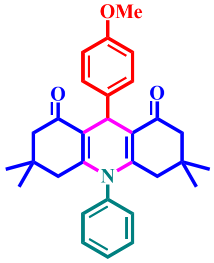

9-(4-methoxyphenyl)-3,3,6,6-tetramethyl-10-phenyl-3,4,6,7,9,10-hexahydroacridine-1,8(2H,5H)-dione (11p).

Yellow solid, m.p. 218 °C. ^{1}H NMR (300 MHz, $CDCl_3$): δ (ppm): 0.81 (s, 6H, $2CH_3$), 0.83 (s, 6H, $2CH_3$), 1.93 (s, 4H, $2CH_2$), 2.09- (s, 4H, $2CH_2$), 3.79 (s, 3H, OCH_3), 4.87 (s, 1H, CH), 6.96-7.23 (m, 4H, ArH), 7.35-7.58 (m, 5H, ArH), ^{13}C NMR (75 MHz, $CDCl_3$): δ(ppm): 27.2. 28.8, 32.7, 32.9, 33.1, 54.0, 55.8, 116.2, 129.4, 131.5, 137.2, 138.2, 139.9, 152.3, 159.6, 199.8.[7]

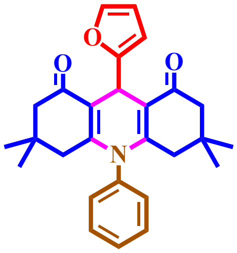

9-(furan-2-yl)-3,3,6,6-tetramethyl-10-phenyl-3,4,6,7,9,10-hexahydroacridine -1,8(2H,5H)-dione (11q).

Light gray solid, m.p. 291 °C. ^{1}H NMR (300 MHz, $CDCl_3$): δ (ppm): 1.44 (s, 6H, $2CH_3$), 1.44 (s, 6H, $2CH_3$), 1.55 (s, 4H, $2CH_2$), 2.91 (s, 4H, $2CH_2$), 4.73 (s, 1H, CH), 6.68 (d, 1H, J = 6.9 Hz Ar-H), 6.72 (d, 1H, J = 6.9 Hz, Ar-H), 6.82 (d, 1H, J = 6.6 Hz, Ar-H), 7.01-7.27 (m, 5H, Ar-H). ^{13}C NMR (75 MHz, $CDCl_3$): δ(ppm): 27.1, 31.6, 45.1, 55.9, 100.1, 153.4, 194.6.[8]

9-(3-chlorophenyl)-3,3,6,6-tetramethyl-10-phenyl-3,4,6,7,9,10-hexahydroacridine-1,8(2H,5H)-dione (11r).

White solid, m.p. 248 °C. ^{1}H NMR (300 MHz, $CDCl_3$): δ (ppm): 0.78 (s, 6H, $2CH_3$), 0.82 (s, 6H, $2CH_3$), 1.69 (d, J = 16.6 Hz, 2H, CH_2), 2. 21 (d, J = 16.6 Hz, 2H, CH_2), 2.08 (d, J = 14.8 Hz, 2H, CH_2), 2.14 (d, J = 14.8 Hz, 2H, CH_2), 5.21

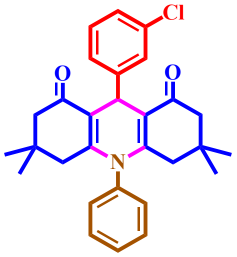

(s, 1H, CH), 7.14 (d, J = 7.6 Hz, 2H, ArH), 7.21 (d, J = 7.6 Hz, 2H, ArH), 7.38 (d, J = 8.6 Hz, 2H, ArH), 7.44 (m, 3H, ArH). ^{13}C NMR (75 MHz, $CDCl_3$): δ(ppm): 26.5, 29.6, 32.4, 33.3, 41.3, 49.8, 112.5, 119.3, 123.6, 129.4, 134.5, 137.5, 146.1, 151.3, 154.0, 199.9.[9]

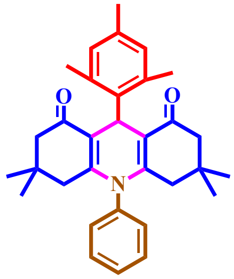

9-mesityl-3,3,6,6-tetramethyl-10-phenyl-3,4,6,7,9,10-hexahydroacridine-1,8(2H,5H)-dione (11s).

White solid, m.p. 312 °C. ^{1}H NMR (300 MHz, $CDCl_3$): δ (ppm): 1.12 (s, 6H, $2CH_3$), 1.29 (s, 6H, $2CH_3$), 2.29 (s, 3H, CH_3), 2.31 (s, 6H, $2CH_3$), 2.42–2.50 (s, 8H, $4CH_2$), 4.31 (s, 1H, CH), 7.12 (s, 2H, Ar-H). 7.32-7.56 (m, 5H, Ar-H). ^{13}C NMR (75 MHz, $CDCl_3$): δ(ppm): 18.6, 18.4. 18.6, 19.9, 26.6, 29.7, 32.3, 32.4, 41.3, 50.0, 112.6, 129.6, 129.7, 136.5, 138.6, 145.6, 150.8, 197.3.[10]

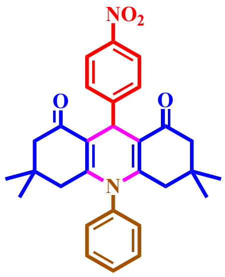

3,3,6,6-tetramethyl-9-(4-nitrophenyl)-10-phenyl-3,4,6,7,9,10-hexahydroacridine-1,8(2H,5H)-dione (11t).

Yellow solid, m.p. 265 °C. ^{1}H NMR (300 MHz, $CDCl_3$): δ (ppm): 0.74 (s, 6H, $2CH_3$), 0.92 (s, 6H, $2CH_3$), 1.35 (d, J = 14.8 Hz, 2H, CH_2), 2.08 (d, J = 14.8 Hz, 2H, CH_2), 2.12 (d, J = 12.4 Hz, 2H, CH_2), 2.18 (d, J = 12.4 Hz, 2H, CH_2), 5.16 (s, 1H, CH), 7.14 (d, J = 7.2 Hz, 2H, ArH), 7.58 (d, J = 7.2 Hz, 2H, ArH), 7.62 (s, 1H, ArH), 7.68 (d, J = 8.2 Hz, 2H, ArH), 8.16 (d, J = 8.2 Hz, 2H, ArH). ^{13}C NMR (75 MHz, $CDCl_3$): δ(ppm): 24.2, 28.2, 31.6, 36.2, 42.4, 52.3, 113.2, 123.8, 129.2, 130.2, 136.4, 147.8, 151.4, 155.8, 197.8.[11]

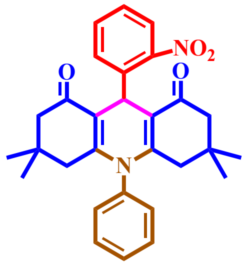

3,3,6,6-tetramethyl-9-(2-nitrophenyl)-10-phenyl-3,4,6,7,9,10-hexahydroacridine-1,8(2H,5H)-dione (11u).

Yellow solid, m.p. 272 °C. ^{1}H NMR (300 MHz, $CDCl_3$): δ (ppm): 0.82 (s, 6H, $2CH_3$), 0.94 (s, 6H, $2CH_3$), 1.78 (d, 2H, J = 16.6 Hz, CH_2), 2.08 (d, 2H, J = 16.4 Hz, CH_2), 2.16 (d, 2H, J = 13.6 Hz, CH_2), 2.28 (d, 2H, J = 13.6 Hz, CH_2), 5.42 (s, 1H, CH), 7.34 (m, 1H, ArH), 7.46 (m, 2H, ArH), 7.64 (m, 3H, ArH), 7.94 (m, 2H, ArH), 8.38 (s, 1H, ArH). ^{13}C NMR (75 MHz, $CDCl_3$): δ(ppm): 26.1, 29.9, 31.2, 32.9, 43.1, 53.4, 112.1, 113.9, 122.1, 127.9, 130.9, 133.6, 138.3, 152.2, 155.2, 198.6.[10]

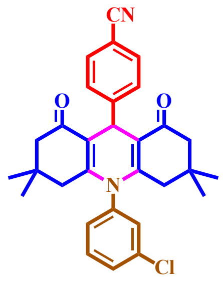

4-(10-(3-chlorophenyl)-3,3,6,6-tetramethyl-1,8-dioxo-1,2,3,4,5,6,7,8,9,10-decahydroacridin-9-yl)benzonitrile (11v).

Yellow solid, m.p. 322 °C. ^{1}H NMR (300 MHz, $CDCl_3$): δ (ppm): 0.72 (s, 6H, $2CH_3$), 0.92 (s, 6H, $2CH_3$), 1.62 (d, 2H, J = 14.5 Hz, CH_2), 2.06 (d, 2H, J = 14.5 Hz, CH_2), 2.11 (d, 2H, J = 12.2 Hz, CH_2), 2.24 (d, 2H, J = 12.2 Hz, CH_2), 5.28 (s, 1H, CH), 7.62 (d, 2H, J = 6.0 Hz, ArH), 7.58 (d, 2H, J = 6.0 Hz, ArH), 7.42 (d, 2H, J = 6.4 Hz, ArH), 7.49 (d, J = 6.4 Hz, 1H, ArH), 7.14 (s, 1H, ArH). ^{13}C NMR (75 MHz, $CDCl_3$): δ(ppm): 24.2, 29.8, 30.8, 32.2, 42.4, 52.4, 111.2, 113.8, 121.4, 127.6, 130.7, 132.7, 137.6, 150.2, 154.1, 195.2.[2]

4-(10-(2-ethyl-6-methylphenyl)-3,3,6,6-tetramethyl-1,8-dioxo-1,2,3,4,5,6,7,8,9,10-decahydroacridin-9-yl)benzonitrile (11w).

Light brown solid, m.p. 313 °C. ^{1}H NMR (300 MHz, $CDCl_3$): δ (ppm): 0.70 (s,

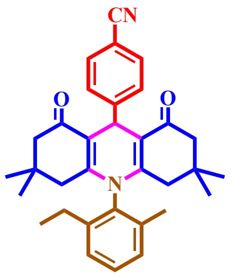

6H, $2CH_3$), 0.90 (s, 6H, $2CH_3$), 1.22 (t, 3H, J = 18.4 Hz, CH_3), 1.80 (d, 2H, J = 17.4 Hz, CH_2), 1.92 (q, 2H, J = 18.4 Hz, CH_2), 2.01 (d, 2H, J = 16.0 Hz, CH_2), 2.19 (d, 4H, J= 16.6 Hz, CH_2), 2.24 (s, 3H, CH_2), 5.10 (s, 1H, CH), 7.48-7.52 (m, 4H, Ar-H), 7.70-7.76 (m, 3H, Ar-H). ^{13}C NMR (75 MHz, $CDCl_3$): δ(ppm): 16.4, 22.7, 26.6, 29.6, 32.5, 33.4, 41.4, 48.7, 49.9, 109.1, 112.6, 119.4, 129.2, 130.5, 132.4, 134.5, 137.6, 151.2, 151.9, 197.3.[12]

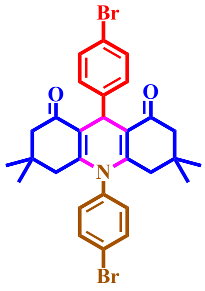

9,10-bis(4-bromophenyl)-3,3,6,6-tetramethyl-3,4,6,7,9,10-hexahydroacridine-1,8(2H,5H)-dione (11x).

White solid, m.p. 298 °C. ^1H NMR (300 MHz, $CDCl_3$): δ (ppm): 0.73 (s, 6H, $2CH_3$), 0.89 (s, 6H, $2CH_3$), 1.82 (d, 2H, J = 18.0 Hz, CH_2), 2.02 (d, 2H, J = 18.0 Hz, CH_2), 2.56-2.19 (m, 4H, $2CH_2$), 5.03 (s, 1H, CH), 7.47-7.44 (m, 4H, ArH), 7.67-7.58 (m, 4H, ArH). ^{13}C NMR (75 MHz, $CDCl_3$): δ(ppm): 26.6, 29.7, 32.3, 32.4, 41.4, 49.4, 112.9, 119.2, 129.9, 130.3, 130.5, 131.2, 138.8, 146.1, 151.0, 195.5.[9]

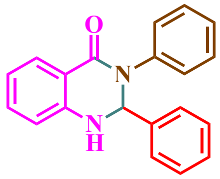

2,3-diphenyl-2,3-dihydroquinazolin-4(1H)-one (12a).

Yellow solid, m.p. 205 °C. ^1HNMR (300 MHz, $CDCl_3$): δ (ppm): 4.74 (s, 1H, CH), 6.06 (s, 1H, NH), 6.62 (d, 1H, J = 7.5 Hz, ArH), 6.89 (t, 1H, J = 7.5 Hz, ArH), 7.13−7.20 (m, 6H, ArH), 7.27−7.31 (m, 3H, ArH), 8.03 (dd, 1H, J = 8.0, 1.5 Hz, ArH). ^{13}C NMR (75 MHz, $CDCl_3$): δ(ppm): 74.6, 114.8, 116.9, 119.5, 123.8, 126.7, 126.8, 127.3, 128.6, 129.0, 129.7, 133.8, 138.5, 139.8, 140.6, 145.2, 163.1.[13]

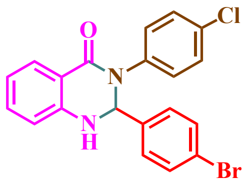

2-(4-bromophenyl)-3-(4-chlorophenyl)-2,3-dihydroquinazolin-4(1H)-one (12b).

Yellow solid, m.p. 217 °C. ^1H NMR (300 MHz, $CDCl_3$): δ (ppm): 4.97 (s, 1H, CH), 6.03 (s, 1H, NH), 6.62 (d, 1H, J = 8.0 Hz, ArH), 6.88 (t, 1H, J = 7.5 Hz, ArH), 7.11 (t, 1H, J = 7.5 Hz, ArH), 7.17−7.20 (m, 3H, ArH), 7.27−7.31 (m, 2H, ArH), 7.38 (d, 1H, J = 8.0 Hz, ArH), 7.49 (s, 1H, ArH), 8.00 (d, 1H, J = 7.0 Hz, ArH). ^{13}C NMR (75 MHz, $CDCl_3$): δ(ppm): 73.9, 115.1, 116.9, 119.9, 122.8, 125.4, 126.8, 126.9, 129.1, 129.9, 130.1, 130.3, 132.1, 134.0, 140.4, 142.3, 144.9, 162.9.[14]

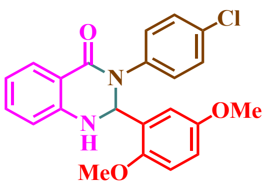

3-(4-chlorophenyl)-2-(2,5-dimethoxyphenyl)-2,3-dihydroquinazolin-4(1H)-one (12C).

White solid, m.p. 249 °C. ^1H NMR (300 MHz, $CDCl_3$): δ (ppm): 3.85 (s, 3H, OCH_3), 3.88 (s, 3H, OCH_3), 5.22 (s, 1H, CH), 6.30 (s, 1H, NH), 6.52 (d, 1H, J = 8.0 Hz, ArH), 6.81 (t, 1H, J = 7.5 Hz, ArH), 6.88 (d, 1H, J = 7.5 Hz, ArH), 6.96 (t, 1H, J = 8.0 Hz, ArH), 7.05 (d, 1H, J = 7.5 Hz, ArH), 7.18−7.22 (m, 2H, ArH), 7.30−7.34 (m, 2H, ArH), 8.01 (d, 1H, J = 7.0 Hz, ArH). ^{13}C NMR (75 MHz, $CDCl_3$): δ(ppm): 49.0, 55.2, 74.4, 113.9, 114.3, 114.6, 116.9, 119.4, 120.6, 126.9, 128.1, 129.0, 129.5, 132.0, 133.7, 136.5, 137.9, 145.4, 159.9, 163.2.[15]

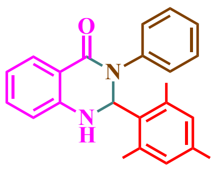

2-mesityl-3-phenyl-2,3-dihydroquinazolin-4(1H)-one (12d).

White solid, m.p. 263 °C. ^1H NMR (300 MHz, $CDCl_3$): δ (ppm): 2.75 (s, 3H, CH_3), 2.82 (s, 3H, CH_3), 2.96 (s, 3H, CH_3), 6.02 (s, 1H, CH), 6.64 (s, 1H, NH), 6.70 (t, 1H, J = 7.5 Hz, ArH), 6.78 (d, 2H, J = 9.0 Hz, ArH), 6.83 (dd, 1H, J = 8.0, 1.5 Hz, ArH), 6.87−6.92 (m, 3H, ArH), 7.05 (d, 2H, J = 9.0 Hz, ArH), 7.31 (td, 1H, J = 8.0, 1.5 Hz, ArH), 8.00 (dd, 1H, J = 8.0, 1.5 Hz, ArH). ^{13}C NMR (75

MHz, $CDCl_3$): δ(ppm): 25.4, 27.8, 28.6, 75.0, 109.9, 110.7, 114.2, 114.5, 116.7, 119.5, 119.8, 128.7, 129.0, 132.2, 133.2, 133.7, 145.7, 149.1, 149.5, 158.2, 163.0. [16]

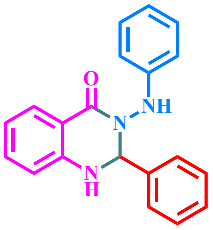

2-phenyl-3-(phenylamino)-2,3-dihydroquinazolin-4(1H)-one (13a)

Yellow solid, m.p. 197 °C. ^{1}H NMR (300 MHz, $CDCl_3$): δ (ppm): 5.89 (s, 1H, CH), 6.68–6.80 (m, 3H, ArH), 6.84 (d, 2H, J = 7.7, ArH), 7.17 (t, 2H, J = 7.4, ArH), 7.25–7.37 (m, 4H, ArH), 7.42 (d, 2H, J = 6.4, ArH), 7.63 (d, 1H, J = 7.3, ArH), 7.65 (s, 1H, NH), 8.38 (s, 1H, NH). ^{13}C NMR (75 MHz, $CDCl_3$): δ(ppm): 74.0, 112.8, 114.1, 115.0, 117.8, 119.7, 126.8, 128.0, 128.7, 129.3, 134.2, 141.2, 147.3, 148.3, 163.0.[17]

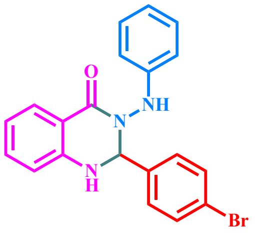

2-(4-bromophenyl)-3-(phenylamino)-2,3-dihydroquinazolin-4(1H)-one (13b).

Yellow solid, m.p. 182 °C. ^{1}H NMR (300 MHz, $CDCl_3$): δ (ppm): 5.91 (s, 1H, CH), 6.69–6.84 (m, 5H, ArH), 7.16 (t, 2H, J = 7.5, ArH), 7.26 (t, 1H, J = 7.0, ArH), 7.40 (d, 2H, J = 8.3, ArH), 7.53 (d, 2H, J = 8.4, ArH), 7.62 (d, 1H, J = 8.3, ArH), 7.64 (s, 1H, NH), 8.39 (s, 1H, NH). ^{13}C NMR (75 MHz, $CDCl_3$): δ(ppm): 73.4, 112.8, 114.6, 115.1, 118.0, 119.7, 122.0, 128.0, 129.1, 129.3, 131.6, 134.3, 140.5, 147.1, 148.1, 164.0.[18]

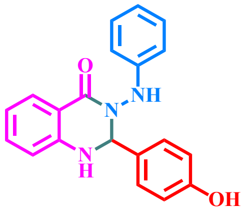

2-(4-hydroxyphenyl)-3-(phenylamino)-2,3-dihydroquinazolin-4(1H)-one (13c).

Yellow solid, m.p. 166 °C. ^{1}H NMR (300 MHz, $CDCl_3$): δ (ppm): 6.08 (s, 1H, CH), 6.66–6.89 (m, 7H, ArH), 7.10–7.22 (m, 1H, NH, 4H, ArH), 7.67 (d, 1H, J = 7.7, ArH), 8.29 (s, 1H, NH), 10.00 (s, 1H, OH). ^{13}C NMR (75 MHz, $CDCl_3$): δ(ppm): 69.4, 112.7, 114.4, 115.3, 115.9, 117.6, 119.1, 119.7, 126.5, 126.7, 127.8, 129.4, 129.7, 134.0, 147.1, 148.1, 155.1, 165.5.[19]

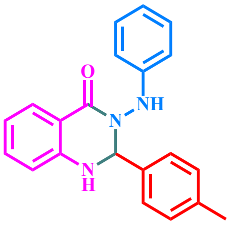

3-(phenylamino)-2-(p-tolyl)-2,3-dihydroquinazolin-4(1H)-one (13d).

Yellow solid, m.p. 193 °C. ^{1}H NMR (300 MHz, $CDCl_3$): δ (ppm): 2.24 (s. 1H, CH_3), 5.92 (s, 1H, CH), 6.71–6.84 (m, 5H, ArH), 7.16 (t, 2H, J = 7.8, ArH), 7.29 (d, 1H, J = 8.5, ArH), 7.40 (d, 2H, J = 8.5, ArH), 7.56 (d, 2H, J = 78.5, ArH), 7.62 (s, 1H, ArH), 7.64 (s, 1H, NH), 8.39 (s, 1H, NH). ^{13}C NMR (75 MHz, $CDCl_3$): δ(ppm): 21.1, 73.8, 112.8, 114.7, 115.0, 117.8, 119.6, 126.7, 127.9, 129.2, 129.3, 134.2, 138.0, 138.2, 147.3, 147.3, 148.3, 163.1.[20]

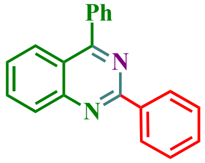

2,4-diphenylquinazoline (14a).

Light brown solid, m.p. 116 °C. ^{1}H NMR (300 MHz, $CDCl_3$): δ (ppm): 7.52–7.61 (m, 7H, ArH), 7.87–7.91 (m, 3H, ArH), 8.15 (t, 2H, J = 8.4 Hz, ArH), 8.69 (d, 2H, J = 8.4 Hz, ArH). ^{13}C NMR (75 MHz, $CDCl_3$): δ(ppm): 121.7, 126.9, 127.1, 128.4, 128.6, 128.7, 129.2, 129.9, 130.2, 130.5, 133.6, 137.7, 138.2, 152.0, 160.3, 168.3.[21]

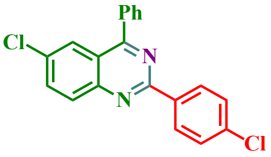

6-chloro-2-(4-chlorophenyl)-4-phenylquinazoline (14b).

White solid, m.p. 189 °C. ^{1}H NMR (300 MHz, $CDCl_3$): δ (ppm): 7.17–7.21 (m, 2H, ArH), 7.52–7.62 (m, 3H, ArH), 7.86–7.90 (m, 3H, ArH), 8.12 (d, 2H, J = 4.0 Hz, ArH), 8.70 (dd, 2H, J = 5.6, 8.4 Hz, ArH). ^{13}C NMR (75 MHz, $CDCl_3$): δ(ppm): 115.4, 121.6, 126.9, 128.6, 129.1, 130.0, 130.2, 130.7, 130.9, 133.6, 134.4, 137.6, 152.0, 159.3, 163.4.[22]

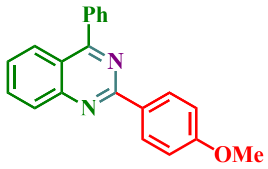

2-(4-methoxyphenyl)-4-phenylquinazoline (14c).
White solid, m.p. 161 °C. ^{1}H NMR (300 MHz, $CDCl_3$): δ(ppm): 3.86 (s, 3H, OCH_3), 7.05 (d, 2H, J = 8.0 Hz, ArH), 7.42–7.50 (m, 4H, ArH), 7.78–7.84 (m, 3H, ArH), 8.06–8.12 (m, 2H, ArH), 8.62 (d, 2H, J = 8.0 Hz, ArH). ^{13}C NMR (75 MHz, $CDCl_3$): δ(ppm): 55.5, 114.0, 121.7, 126.8, 127.1, 128.5, 128.7, 129.2, 130.2, 130.4, 131.9, 133.4, 138.3, 152.1, 160.2, 161.3, 167.7.[23]

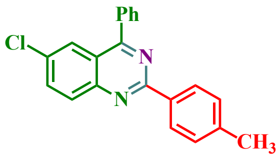

6-chloro-4-phenyl-2-(*p*-tolyl)quinazoline (14d).
Yellow solid, m.p. 144°C. ^{1}HNMR (300 MHz, $CDCl_3$): δ (ppm): 2.24 (s, 3H, CH_3), 7.39–7.53 (m, 7H, ArH), 7.69 (d, 1H, J = 8.4 Hz, ArH), 7.88 (t, 1H, J = 7.6 Hz, ArH), 8.16 (d, 1H, J = 8.4Hz, ArH), 8.66 (d, 2H, J = 8.0 Hz, ArH). ^{13}C NMR (75 MHz, $CDCl_3$): δ(ppm): 19.0, 121.6, 124.6, 126.0, 126.1, 127.5, 127.7, 128.0, 128.2, 128.6, 129.5, 129.7, 132.7, 135.4, 135.9, 137.2, 150.4, 159.3, 168.8.[24]

References

1- Zarei, Z., & Akhlaghinia, B. (2017). Zn II doped and immobilized on functionalized magnetic hydrotalcite (Fe 3 O 4/HT-SMTU-Zn II): a novel, green and magnetically recyclable bifunctional nanocatalyst for the one-pot multi-component synthesis of acridinediones under solvent-free conditions. *New Journal of Chemistry*, *41*(24), 15485-15500.

2- Pamuk, H., Aday, B., Şen, F., & Kaya, M. (2015). Pt NPs@ GO as a highly efficient and reusable catalyst for one-pot synthesis of acridinedione derivatives. *RSC Advances*, *5*(61), 49295-49300.

3- Işık, A., Aday, B., Ulus, R., & Kaya, M. (2015). One-pot, facile, highly efficient, and green synthesis of acridinedione derivatives using vitamin B1. *Synthetic Communications*, *45*(24), 2823-2831.

4- Maleki, B., Mofrad, A. V., Tayebee, R., Khojastehnezhad, A., Alinezhad, H., & Seresht, E. R. (2017). One-Pot Synthesis of 1, 4-Dihydropyridine Derivatives Catalyzed by Silica-Coated Magnetic NiFe 2 O 4 Nanoparticles-Supported H 14 [NaP 5 W 30 O 110]. *Russian Journal of General Chemistry*, *87*(12), 2922-2929.

5- Dehbalaei, M. G., Foroughifar, N., Pasdar, H., & Khajeh-Amiri, A. (2018). N-Propyl benzoguanamine sulfonic acid supported on magnetic Fe 3 O 4 nanoparticles: a novel and efficient magnetically heterogeneous catalyst for the synthesis of 1, 8-dioxo-decahydroacridine derivatives. *New Journal of Chemistry*, *42*(1), 327-335.

6- Khazaei, A., Moosavi-Zare, A. R., Mohammadi, Z., Khakyzadeh, V., & Afsar, J. (2016). Nano-TiO2 as an Efficient Catalyst for Tandem Knoevenagel–Michael-Cyclocondensation Reaction of Dimedone with Aromatic Aldehydes and Ammonium Acetate or Aromatic Amines under Solvent-free Conditions. *Journal of the Chinese Chemical Society*, *63*(2), 165-170.

7- Sughanya, V., & Sureshbabu, N. (2012). 10-Benzyl-9-(4-ethoxyphenyl)-3, 3, 6, 6-tetramethyl-3, 4, 6, 7, 9, 10-hexahydroacridine-1, 8 (2H, 5H)-dione. *Acta Crystallographica Section E: Structure Reports Online*, *68*(9), o2755-o2755.

8- Alam, M. M., Mubarak, A. T., Assiri, M. A., Ahmed, S. M., & Fouda, A. M. (2019). A facile and efficient synthesis of 1, 8-dioxodecahydroacridines derivatives catalyzed by cobalt–alanine metal complex under aqueous ethanol media. *BMC chemistry*, *13*(1), 1-10.

9- Shukla, D. V., Kumar, A., Srivastava, A. K., Misra, N., & Brahmachari, G. (2017). Spectral (FT-IR, NMR) Analyses, Molecular Structures, and Chemical Bonding of Two Hexahydroacridine-1, 8 (2 H, 5 H)-dione Derivatives: A Comparative Quantum Chemical Study. *Polycyclic Aromatic Compounds*, *37*(5), 426-441.

10- Abdelhamid, A. A., Mohamed, S. K., & Simpson, J. (2014). Crystal structure of 9-(3-bromo-5-chloro-2-hydroxyphenyl)-10-(2-hydroxyethyl)-3, 3, 6, 6-tetramethyl-3, 4, 6, 7, 9, 10-hexahydroacridine-1, 8 (2H, 5H)-dione. *Acta Crystallographica Section E: Structure Reports Online*, *70*(8), 44-47.

11- Mohammadi Ziarani, G., Rahimifard, M., Badiei, A., & Abolhasani Soorki, A. (2016). Fast one-pot synthesis of 1, 8-dioxo-decahydroacridine derivatives using sulfonic acid functionalized LUS-1 and the study on their antimicrobial activities. *Iranian Journal of Catalysis*, *6*(4), 369-375.

12- Khalilov, A. N., Abdelhamid, A. A., Gurbanov, A. V., & Ng, S. W. (2011). 9-(5-Bromo-2-hydroxyphenyl)-10-(2-hydroxypropyl)-3, 3, 6, 6-tetramethyl-1, 2, 3, 4, 5, 6, 7, 8, 9, 10-decahydroacridine-1, 8-dione. *Acta Crystallographica Section E: Structure Reports Online*, *67*(5), o1146-o1146.

13- Zhang, Z. H., Lü, H. Y., Yang, S. H., & Gao, J. W. (2010). Synthesis of 2, 3-dihydroquinazolin-4 (1 H)-ones by three-component coupling of isatoic anhydride, amines, and aldehydes catalyzed by magnetic Fe3O4 nanoparticles in water. *Journal of combinatorial chemistry*, *12*(5), 643-646.

14- Mohammadi Ziarani, G., Kazemi Asl, Z., Gholamzadeh, P., Badiei, A., & Afshar, M. (2017). The use of SrFe12O19 magnetic nanoparticles as an efficient catalyst in the modified Niementowski reaction. *Applied Organometallic Chemistry*, *31*(12), e3830.

15- Dabiri, M., Salehi, P., Otokesh, S., Baghbanzadeh, M., Kozehgary, G., & Mohammadi, A. A. (2005). Efficient synthesis of mono-and disubstituted 2, 3-dihydroquinazolin-4 (1H)-ones using KAl (SO4) 2· 12H2O as a reusable catalyst in water and ethanol. Tetrahedron Letters, 46(36), 6123-6126.

16- Wu, J., Du, X., Ma, J., Zhang, Y., Shi, Q., Luo, L., ... & Hu, D. (2014). Preparation of 2, 3-dihydroquinazolin-4 (1 H)-one derivatives in aqueous media with β-cyclodextrin-SO 3 H as a recyclable catalyst. *Green Chemistry*, *16*(6), 3210-3217.

17- Ma, Y., Ren, D., Zhang, J., Liu, J., Zhao, J., Wang, L., & Zhang, F. (2015). Synthesis, antibacterial activities evaluation, and docking studies of some 2-substituted-3-(phenylamino)-dihydroquinazolin-4 (1H)-ones. Tetrahedron Letters, 56(27), 4076-4079.

18- Hajjami, M., Ghorbani, F., & Yousofvand, Z. (2017). Copper (I) complex of 1, 3-DimethylBarbituric acid modified SBA-15 and its catalytic role for the synthesis of 2, 3-Dihydroquinazolin-4 (1H)-ones and Imidazoles. *Applied Organometallic Chemistry*, *31*(12), e3843.

19- Tamoradi, T., Ghadermazi, M., & Ghorbani-Choghamarani, A. (2018). Synthesis of polyhydroquinoline, 2, 3-dihydroquinazolin-4 (1H)-one, sulfide and sulfoxide derivatives catalyzed by new copper complex supported on MCM-41. *Catalysis Letters*, *148*(3), 857-872.

20- Gao, L., Ji, H., Rong, L., Tang, D., Zha, Y., Shi, Y., & Tu, S. (2011). An efficient synthesis of 2, 3-dihydroquinazolin-4 (1H)-one derivatives under catalyst-free and solvent-free conditions. *Journal of Heterocyclic Chemistry*, *48*(4), 957-960.

21- Ferrini, S., Ponticelli, F., & Taddei, M. (2007). Convenient synthetic approach to 2, 4-disubstituted quinazolines. *Organic letters*, *9*(1), 69-72.

22- Sarma, R., & Prajapati, D. (2011). Microwave-promoted efficient synthesis of dihydroquinazolines. *Green chemistry*, *13*(3), 718-722.

23- Fatehi, A., Ghorbani-Vaghei, R., Alavinia, S., & Mahmoodi, J. (2020). Synthesis of Quinazoline Derivatives Catalyzed by a New Efficient Reusable Nanomagnetic Catalyst Supported with Functionalized Piperidinium Benzene-1, 3-Disulfonate Ionic Liquid. *ChemistrySelect*, *5*(3), 944-951.
24- Eidi, E., Kassaee, M. Z., Nasresfahani, Z., & Cummings, P. T. (2018). Synthesis of quinazolines over recyclable Fe3O4@ SiO2-PrNH2-Fe3+ nanoparticles: A green, efficient, and solvent-free protocol. *Applied Organometallic Chemistry*, *32*(12), e4573.
